# The Importance of Lateral Connections in the Parietal Cortex for Generating Motor Plans

**DOI:** 10.1371/journal.pone.0134669

**Published:** 2015-08-07

**Authors:** Derrik E. Asher, Nicolas Oros, Jeffrey L. Krichmar

**Affiliations:** 1 Department of Cognitive Sciences, University of California Irvine, Irvine, California, United States of America; 2 Department of Computer Science, University of California Irvine, Irvine, California, United States of America; Plymouth University, UNITED KINGDOM

## Abstract

Substantial evidence has highlighted the significant role of associative brain areas, such as the posterior parietal cortex (PPC) in transforming multimodal sensory information into motor plans. However, little is known about how different sensory information, which can have different delays or be absent, combines to produce a motor plan, such as executing a reaching movement. To address these issues, we constructed four biologically plausible network architectures to simulate PPC: 1) feedforward from sensory input to the PPC to a motor output area, 2) feedforward with the addition of an efference copy from the motor area, 3) feedforward with the addition of lateral or recurrent connectivity across PPC neurons, and 4) feedforward plus efference copy, and lateral connections. Using an evolutionary strategy, the connectivity of these network architectures was evolved to execute visually guided movements, where the target stimulus provided visual input for the entirety of each trial. The models were then tested on a memory guided motor task, where the visual target disappeared after a short duration. Sensory input to the neural networks had sensory delays consistent with results from monkey studies. We found that lateral connections within the PPC resulted in smoother movements and were necessary for accurate movements in the absence of visual input. The addition of lateral connections resulted in velocity profiles consistent with those observed in human and non-human primate visually guided studies of reaching, and allowed for smooth, rapid, and accurate movements under all conditions. In contrast, Feedforward or Feedback architectures were insufficient to overcome these challenges. Our results suggest that intrinsic lateral connections are critical for executing accurate, smooth motor plans.

## Introduction

Classically, the posterior parietal cortex (PPC) has been assessed as an association region that combines information from multiple sensory modalities to generate a fused representation of physical space [1–3]. Since then, substantial evidence has indicated that the PPC is necessary for the sensorimotor transformation of multiple pieces of sensory input into a motor plan [4–16], and important for aspects of movement [17–26]. While there is plenty of evidence supporting the PPC’s role in sensorimotor transformations, little is known about how intermittent sensory information or delays are integrated and how that sensory information is utilized to produce a complex motor plan.

Areas such as the PPC, can execute accurate motor plans in the absence of sensory input. PPC neural activation from sensory and motor components has been separated with a memory guided (MG) task [17, 27]. Typically in a MG task, an animal is cued to the location for a movement by a briefly flashed visual stimulus and must withhold the response for > = 1s until a go signal is provided. PPC neurons show sequences of activation for the cue, delay, and movement periods, implying that sensory, memory, and motor components are represented [13, 28]. PPC activation sequences observed during the delay period of the MG task (i.e., after the cue extinguished and before the movement started), indicate that PPC neurons encode some representation of the flashed sensory cue [29]. Furthermore, Human and non-human primate experiments involving MG movements have revealed that PPC activation sequences are representative of the direction of a movement plan instead of memory or sensory related neural activation [10, 30, 31]. Therefore, in a MG reaching task, one would expect to observe PPC activation sequences that represent a movement plan in the absence of the visual stimulus, instead of the memory of that previously flashed visual stimulus. The simulation experiments in the current study utilize an MG task void of a delay period. This indicates that all simulation results pertaining to the MG task do not show memory related PPC activity, instead, the MG task data only show PPC activity in the absence of the visual stimulus, which should not be interpreted as memory related neural responses.

Careful experimentation has shown that passive sensory inputs associated with sensorimotor transformations arrive in PPC neural activity with different latencies. Previously, it has been proposed that select sensory inputs require a minimum amount of time due to physiological constraints (i.e., conduction delays from axonal connection lengths) [32, 33]. In neural recording experiments, it was found that select PPC neurons (in monkey) contained significant movement related information from 180ms before movement initiation to 180ms after movement initiation (data binned in 30ms windows). The neurons with optimal lag times (OLTs) > = 0 indicated predictive motor-goal estimates of future sensory inputs, whereas, OLTs < 0 indicated passive sensory feedback with sensory delays. The fact that neurons with negative OLTs were found in each bin [-180ms, -30ms; in 30ms increments] indicates that passive sensory feedback was delayed by as much as 180ms and as little as 30ms [33]. Utilizing this study as the foundation for minimum sensory delays associated with PPC neuronal responses to proprioceptive and visual information, respectively, at least 30ms and 90ms delays can account for biologically realistic latencies. To understand how sensorimotor transformations are represented in PPC, these sensory latencies must be considered.

Models based on computational neuroscience have provided significant understanding towards neural activation connected with sensorimotor transformations for reaching actions [34–41]. Computational models have shown how different reference frames (e.g., head centered and eye centered) can be transformed into movements [40, 41]. Specifically, a modeling experiment showed that potential actions were simultaneously represented in populations of simulated cells in the parieto-frontal network [35]. The results from this study indicated that neural processing associated with action selection (decision-making) and action execution may be performed at the same time within the same brain regions, instead of in a sequence as was previously thought [42–45]. Built upon this work in a recent study [46], a dynamic neural field (DNF) model learned arbitrary sensorimotor associations (motor-goals tied to abstract contextual information) with a reward-driven Hebbian learning algorithm. This set of DNF model simulation experiments, showed how working memory and action selection can dynamically influence the sensorimotor integration process involved with reaching actions. These important modeling studies demonstrated how action selection can be simultaneously processed by brain areas within the parieto-frontal neural network, however, they did not investigate how neural networks with different types of projections combine sensory inputs (sensory integration) with different latencies, to produce a reaching movement.

More closely related to the work presented in this article, a simulation study with three layer neural networks showed that a visual sensory signal could be integrated with abstract contextual rules [34]. In this study, the hidden layer representing the PPC had recurrent connections that were tuned with a modified back-propagation-through-time (BPTT) algorithm to select the correct motor-goal in a visually guided reaching paradigm. Recurrent hidden layer connections in the model served as a type of memory trace that was able to maintain a stable sensory representation of the task in the absence of a visual stimulus. The models did not actually execute the selected actions; instead, the results from these experiments provided theoretical evidence that the integration of sensory and contextual cues in the parietal cortex occurs via a gain-modulation mechanism. This implies that the sensory information is combined with motor and contextual information in the same way that different sensory inputs are combined in parietal brain regions like PRR. Furthermore, their results suggest that the sensory-context integration could be explained by the strong feedback connections from motor output stages (i.e., PMd or M1) that provide the parietal cortex with a high-level executive-like signal.

However, the aforementioned models did not investigate how different projections within the parieto-frontal network contribute to the execution of motor plans that incorporate sensory delays, and the absence of sensory stimuli. Thus, the main goal of the present study is to investigate the sufficiency of different neuroanatomical architectures in constructing motor plans that can cope with sensory delays and the absence of visual input. Specifically, we compared the performance of neurobiologically plausible models in simulation experiments with these architectures: 1) feedforward from sensory input to the PPC projecting to a motor output area (FF), 2) feedforward with the addition of an efference copy (feedback) from the motor area (FB), 3) feedforward with the addition of lateral recurrent connections (lateral) within the PPC (LAT), and 4) feedforward plus feedback and lateral connections (FBLAT). An evolutionary algorithm was used to tune the models in a visually guided reaching task. The models were then tested in a memory guided reaching task, in order to determine if they could cope with the absence of a visual target by preserving its location (via a directional motor plan), and which connections contributed to the mechanisms involved.

## Results

### 2.1 Reaching Tasks

Visually guided (VG) and memory guided (MG) reaching tasks were simulated to test the models’ respective abilities to perform precise movements with and without visual input from a target stimulus. The models were trained on a VG task and tested on a MG task. Although it is not meant to be an exact replica of empirical studies with humans and non-human primates [30, 47–51], these reaching tasks were designed to highlight the mechanisms underlying the construction of a motor plan from multimodal input.

For the simulations in the current study, trials lasted 50 timesteps where each timestep corresponded to 10 milliseconds of real time, indicating the simulation of 500ms trials. Both the VG and MG tasks started with the illumination of the visual target on the 1^st^ timestep of every trial in one of eight possible peripheral positions. For the VG task, the target remained illuminated for all 50 timesteps of the trials (S1 Fig). For the MG task, the visual target extinguished after 5 timesteps (S1 Fig), requiring an agent to maintain an internal representation of the target location (i.e., quickly assemble a directional course of action) in order to perform the task well.

Each trial started with an agent’s central fixation aligned with the hand position centered in space (retino-centric coordinates) indicated by a position [0°, 0°]. Each trial was 50 timesteps designated to simulate 500ms of real-time behavior. The reaching space was constructed in degrees of visual angle from the agent’s central fixation mapped into 2-D Cartesian space (S1 Fig) that was bounded between [-50°, 50°] in both the horizontal and vertical directions. All agents performed 8 trials, a trial for each of the targets located 25° (4 straight: up, down, left, and right) and 35° (4 diagonals) of visual angle from the hand’s initial position in the center of space.

The goal for every trial was to move the hand (end effector) to a target location as fast as possible, and remain at that location for the duration of the trial. It was assumed that motor output dictated the direction and velocity of hand movements. Joint angles and the control of multiple degrees of freedom were not considered in these simulations, nor was the frame of reference or the sensory specific tuning function representing sensory projections onto PPC. The frame of reference was assumed to be retinotopic or gaze centered, which was fixed (static) across all trials, and a cosine tuning curve with slightly different amplitudes was assumed to represent the two sensory specific tuning functions.

### 2.2 Neural Networks

**Fig 1:**
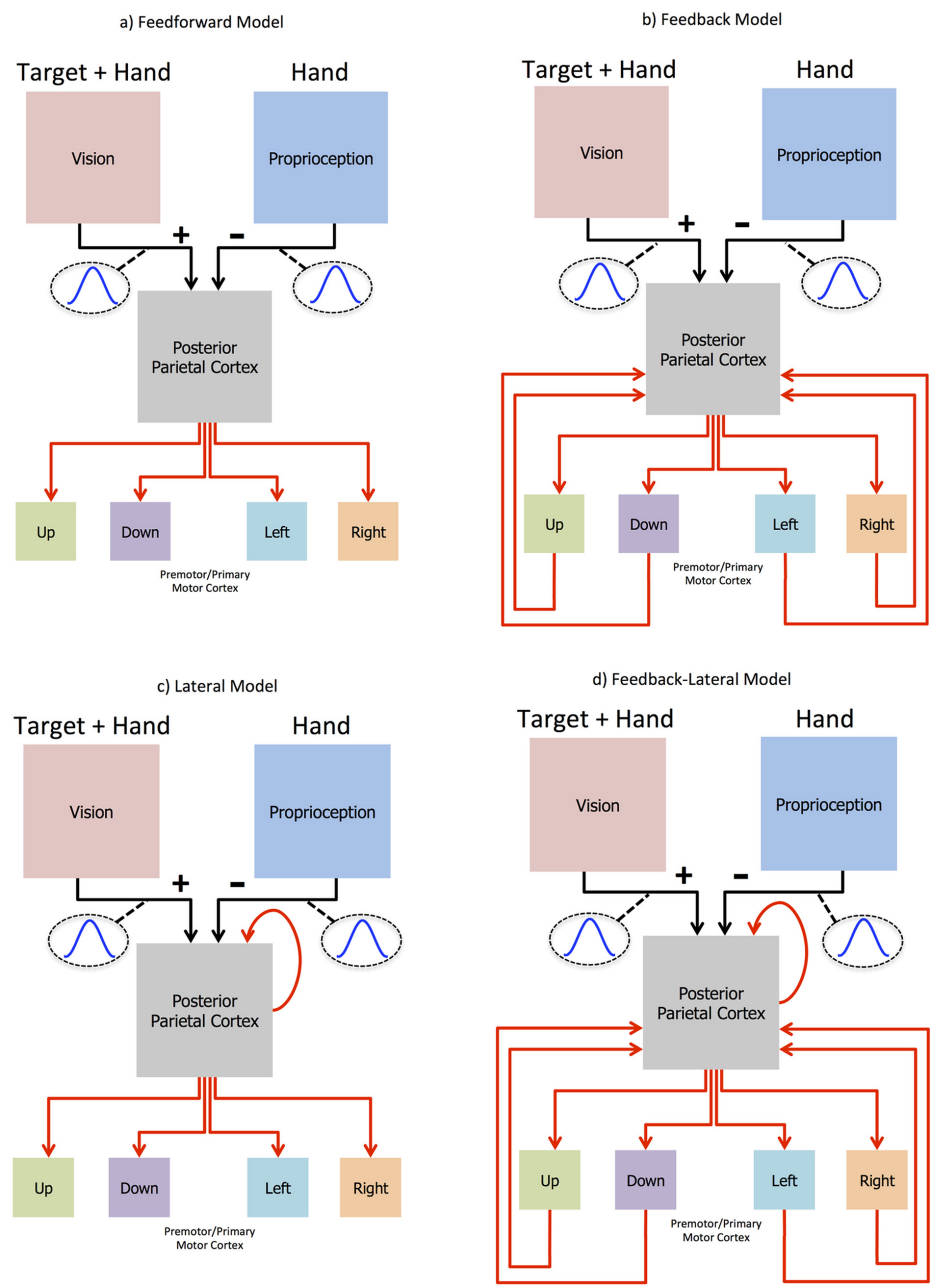
Models. All models have the same general architecture with 121 (11x11) visual neurons (Vision) and 121 (11x11) proprioceptive neurons (Proprioception) feeding forward with opposing 2-D cosine projections (Vision: positive projection with a tuning width of 7 pixels and a maximum amplitude of 2; Proprioception: negative projection with a tuning width of 9 pixels and a maximum amplitude of 4) into 121 (11x11) posterior parietal cortex (PPC) neurons. The visual projections into PPC neurons had half the strength (amplitude) of the proprioceptive projection, to balance out the visual and proprioceptive inputs when the two visual (Hand + Target) representations aligned with the proprioceptive (Hand) representation. The PPC neurons feed forward into the 4 neurons contained within the premotor/primary motor (PMd/M1) cortical region of the network. The black arrows indicate non-evolvable 2-D cosine connectivity between the respective regions. The red arrows indicate evolvable fully connected weights. a) The Feedforward model with fully connected evolvable weights projecting from the PPC to the 4 PMd/M1 neurons. b) The FB model is the same as the FF model along with fully connected evolvable weights projecting back to the PPC from the 4 PMd/M1 neurons. c) The LAT model is the same as the FF model along with fully connected evolvable recurrent weights projecting from the PPC neurons back to themselves. d) The FBLAT model is a combination of the FB and LAT models.

Four biologically plausible neural network architectures (FF, FB, LAT, and FBLAT) were constructed to perform the reaching tasks. All models had the same neural layers (Fig 1; Vision, Proprioception, Posterior Parietal Cortex, and Premotor/Primary Motor Cortex) with the same amount of neurons (11x11, 11x11, 11x11, 1x4 respectively). Topographic cosine tuning curves projected from the visual (Fig 1; Vision) and proprioceptive (Fig 1; Proprioception) input neurons to the posterior parietal cortex (PPC) neurons. The PPC neurons were fully connected to each of the (Up, Down, Left, and Right) premotor/primary motor (PMd/M1) cortex neurons. The models differed in their projections to the PPC neurons. 1) The Feedforward (FF) model’s PPC neurons only received sensory input from vision and proprioception (Fig 1A). 2) The Feedback (FB) model’s PPC neurons received the same sensory input as the FF model, along with fully connected feedback from the PMd/M1 neurons (Fig 1B). 3) The lateral (LAT) model’s PPC neurons received the same sensory input as the FF model, along with fully connected recurrent lateral inputs from all PPC neurons, including self-connections (Fig 1C). 4) The Feedback-Lateral (FBLAT) model’s PPC neurons received the same sensory input as the FF model, along with feedback input like the FB model, and lateral input like the LAT model (Fig 1D).

Based on evidence from empirical studies [52], we imposed sensory delays for visual and proprioceptive inputs. Mulliken and colleagues indicated that the proprioceptive latency could not be less than 30ms and that the visual latency could not be less than 90ms. To implement these minimum latencies into our models, we provided our PPC neurons with 3 timestep delayed proprioceptive hand location information and 9 timestep delayed visual information. This indicates that the models did not receive the relevant visual sensory information from the target or hand locations (in 2-D reaching space) until the 9^th^ timestep of a trial in both tasks, leaving models blind to the goal of the task for the first 1/5^th^ of each trial. Proprioceptive input was received by PPC neurons upon the 3^rd^ timestep of a trial, however it did not indicate the location of the target stimulus or the goal of the task.

### 2.3 Evolutionary Algorithm

An evolutionary algorithm (EA) was used to search the parameter space of the neural network models. The free parameters and their value ranges for the neural networks were the strength of the connections [-1.0, 1.0], the biases [-5.0, 5.0] and the gains [0.1, 10] for neurons in PMd/M1 and PPC (see Eq 2 in section 4.2 PPC and PMd/M1 Neural Firing). These free parameters were evolved using an EA over 25000 generations. The EA utilized in our simulations was “mu comma lambda” (μ, λ), an evolution strategy that included elitism, mutation, and roulette crossover [53].

Weights connecting PPC to PMd/M1 (feedforward connections), PMd/M1 to PPC (feedback connections), and intrinsic PPC to PPC (lateral connections), were subject to change based on the evolutionary algorithm described in section 4.4 (see section 4.4 Evolutionary Algorithm). The weights were randomly initialized within the range of [-1.0, 1.0], and could not change (non-plastic) after the 25000 generations of evolution. The FF model had 484 evolvable weights; the FB model had 968 evolvable weights; the LAT model had 15125 evolvable weights, and the FBLAT model had 15609 evolvable weights.

Each of the model architectures was evolved with 100 independent runs of the evolutionary algorithm, generating 100 best agents (population) per architecture. Fitness was based on an agent’s ability to move quickly and accurately to all targets (see Eq 5 in section 4.4 Evolutionary Algorithm). The agents with the lowest fitness values (fitness minimization) of the respective populations (100 agents per model) were considered the ‘champion’ agents, indicating four total champion agents (see also S1 Text; Evolutionary Algorithm section).

### 2.4 Movement Trajectories and Agent Fitness

**Fig 2:**
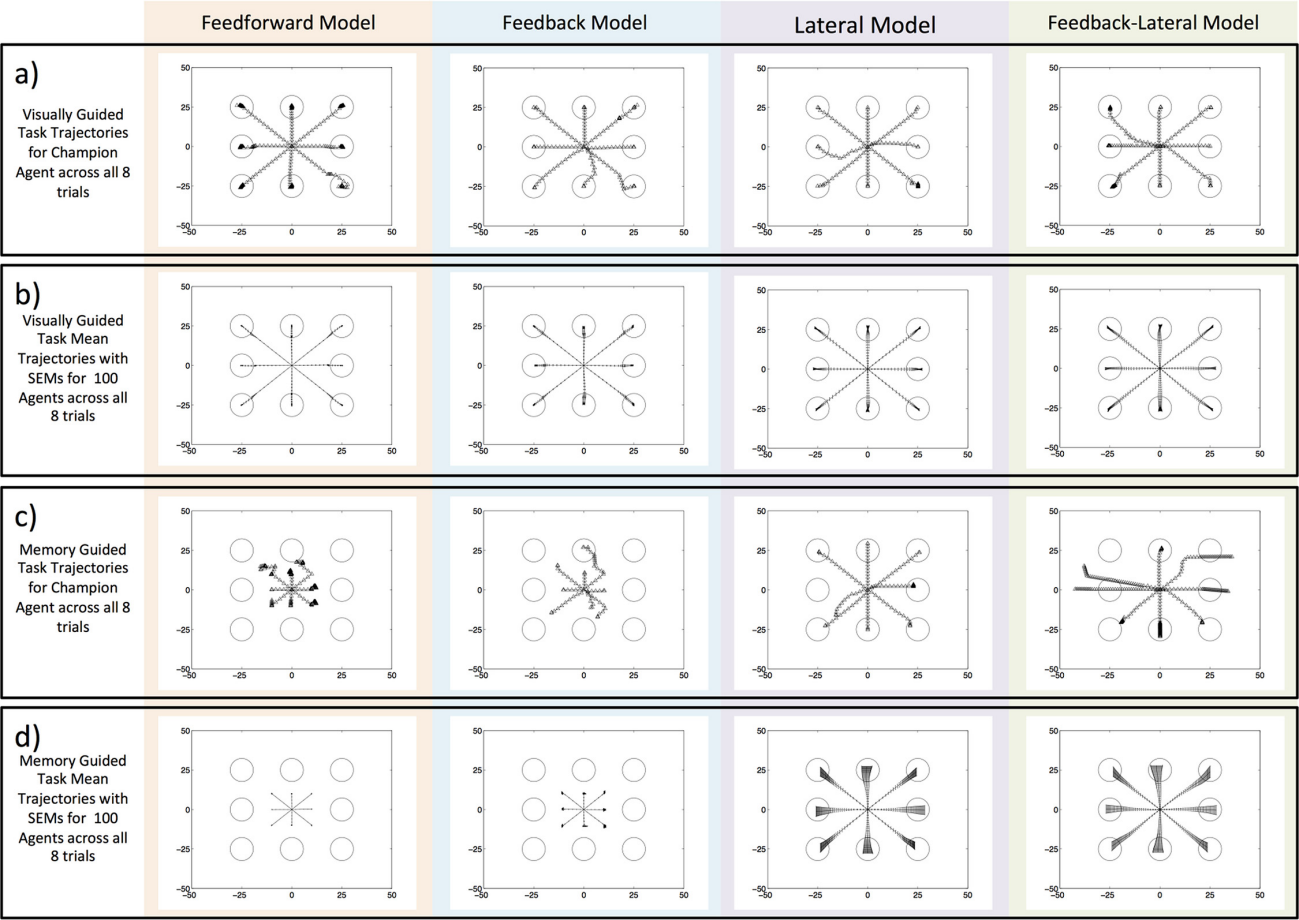
Reaching Trajectories. All plots show the reaching trajectories across all 8 trials with each point representing the hand position at a timestep in 2-D Euclidian space mapped in degrees of visual angle. The circles are centered on the target locations for the 8 trials. The x and y-axes are labeled in degrees of visual angle ranging from [–50, 50] in both the horizontal and vertical directions. The columns represent the data from the four models (from left to right: FF, FB, LAT, and FBLAT). a) The champion agents’ reaching trajectories for the VG task. b) The means and standard error of the means (SEMs) for reaching trajectories at each timestep from the 100 independently evolved best agents (including the champion agent) for the VG task. c) The champion agents’ reaching trajectories for the MG task. d) The population means and SEMs for reaching trajectories at each timestep from the 100 independently evolved best agents (including the champion agent) for the MG task. In a and b, all four models’ performed well on the VG task. In c and d, only the LAT and FBLAT models performed well in the MG task.

In the visually guided task, all agents demonstrated accurate movement, but in the absence of sensory input (memory guided task) more complex connectivity within the parietal cortex was necessary. Specifically, we found that intrinsic connectivity in the simulated PPC was needed for precise reaching actions in the absence of a visual target stimulus. This can be seen in the agents’ movement trajectories (Fig 2), fitness values (S2 and S3 Figs), and average target error (see also S4 Fig and S1 Text; Fitness Comparisons section).

For the movement trajectories shown in Fig 2, each data point represents the hand position in space per timestep across the 8 trials (50 data points per trial). The distance between the data points indicates the rate at which an agent’s hand was moving. All four models’ trajectories in the VG task (Fig 2A and 2B) reflect the excellent performance indicated by fitness (S4 Fig: rows 1, 2; and S2 and S3 Figs) and target error (S4 Fig: rows 3, 4). Models’ champion agent trajectories (Fig 2A) show the absolute path to each target location (8 trials), starting from the center of space. For the VG task, it can be seen that there is not a large difference between the champions’ trajectories for the different architectures (Fig 2A). However, it is interesting to note that the FB, LAT, and FBLAT models (Fig 2A, columns 2, 3, 4) evolved curved trajectories towards some of the target locations, similar to trajectories observed in control subjects [47–49, 51], whereas, the FF model moved with straight paths. This phenomenon is emphasized when inspecting the models’ trajectories for their respective populations (Fig 2B), where the error bars represent the standard error of the mean (SEM). Pairing the movement trajectory data with the respective distributions of fitness values (S3 Fig) provides a metric for the champion agents’ performance (see also S5 Fig and S1 Text; Target Error Comparisons section).

Similarly, the models’ trajectories in the MG task (Fig 2C and 2D) reflect agent performance and highlight the importance of lateral connections in the PPC (S3 and S4 Figs). The different trajectories by the champion agents (Fig 2C) allow for visible confirmation that the FF and FB models (Fig 2C, columns 1, 2) were incapable of performing the MG task, whereas, the LAT and FBLAT models were able to perform this task (Fig 2C and 2D, columns 3, 4).

It can be seen that the differences between the models’ performance in the two tasks emphasizes the importance of lateral connections when conducting reaches to flashed (MG task) target locations (compare Fig 2A to Fig 2C and Fig 2B to Fig 2D). The data show that the absence of a visual target resulted in different behaviors (compare Fig 2B to Fig 2D). Specifically, the LAT model’s champion agent produced near optimal reaches to targets in both the VG and MG tasks (compare column 3 of Fig 2A to column 3 of Fig 2C). Additionally, the FBLAT model’s champion agent was able to move towards the correct target location for the majority of trials in the MG task, but was unable to stop (Fig 2C, column 4).

### 2.5 Velocity Profiles

**Fig 3:**
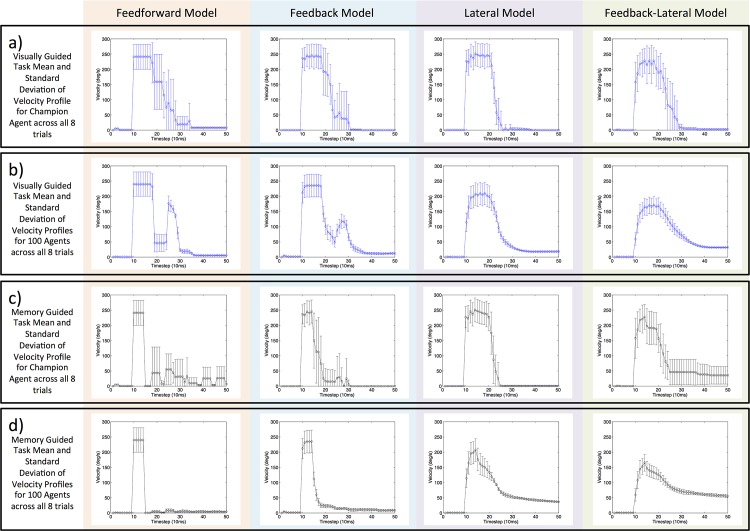
Velocity Profiles. All plots show the average velocity profile across all 8 trials. Every point represents the mean instantaneous velocity per timestep with the error bars showing the standard deviation of velocity across the trials. The x-axes show the timesteps from 1 to 50, representing 10ms per timestep and y-axes show the velocity ranging from 0–300 degrees per second. The columns show the data from the four models with the sensory input weight scale factor (*f*) set to +2 for Vision and -4 for Proprioception (see Eq 1; *f* parameter) representing vector subtraction of the sensory inputs. a) The champion agents’ average velocity profile for the VG task. b) The average velocity profile for all 100 independently evolved best agents (including the champion agent) for the VG task. c) The champion agents’ average velocity profile for the MG task. d) The population averaged velocity profile for all 100 independently evolved best agents (including the champion agent) for the MG task. The LAT and FBLAT models (columns 3 and 4) generated smooth velocity profiles, while the FF and FB models (columns 1 and 2) tended to produce double humped profiles (also see S3, S4 and S6 Figs).

To better understand the dynamics of agents’ movements, average velocity profiles were calculated. The velocity of an agent’s hand movement was determined by the difference between two hand positions at consecutive timesteps, across all timesteps and trials (Fig 3). For the champion agents, the velocity profiles show the average velocity over the 8 trials. For the population of agents, the velocity profiles show the average across the 8 trials for all 100 agents. The error bars represent standard deviations in the respective plots. Critically, the velocity profiles in Fig 3 are set-up to match the movement trajectory plots in Fig 2, indicating that the velocity profiles (Fig 3) are the average rate of hand movement in the trajectory plots (Fig 2).

For the VG task, the velocity profiles (Fig 3A and 3B) provide insight as to how the agents moved towards the target locations and further demonstrate the importance of lateral connections in the PPC. Only LAT and FBLAT models had smooth velocity profiles that accelerated to a maximum velocity and then decelerated until the movement ceased. These velocity profiles are similar to those observed in empirical studies [54–57]. In contrast, the FF and FB models had jagged, and sometimes double humped velocity profiles (Fig 3B, columns 1 and 2), which may have resulted from the specific choice of opposite signs on the sensory input weight values (see Eq 1; *f* parameter), and were selected to demonstrate vector subtraction (i.e., the combination of opposite signs represented by different sensory inputs: positive values for visual weights summed with negative values for proprioceptive weights), similar to what has been shown in empirical studies [58, 59]. This implies that the FF and FB models had difficulties coping with the sensory latencies due to the sensory input weights and compensated by making two sub-movements instead of a single optimized movement like the LAT and FBLAT models.

To further investigate this phenomenon, the LAT and FF models were evolved with a different set of sensory input weight values (Eq 1; *f* parameter set to +4 for both Vision and Proprioception). It was found that the LAT model’s velocity profile remained smooth and single-humped, while the FF model’s profile remained rigid and abrupt, but reduced to a single-hump as well (see S6 Fig). Therefore, the lateral connections proved to be important for smooth velocity profiles when coping with sensory delays, but double-humped velocity profiles appear to be idiosyncratic to the input parameter choice.

Both the FF and FB models started moving at maximum velocity (first hump) immediately after the visual input from the target arrived at the PPC (Fig 3B, columns 1 and 2, timestep 10). Then both models had a sharp decrease in movement rate once the proprioceptive representation of the hand moved towards the target (Fig 3B, columns 1 and 2, timestep 19) and the visual representation had not moved yet (due to different sensory latencies). The second hump in the velocity profiles occurred when the visual representation of the hand caught up to the proprioceptive representation of the hand, resulting in movement towards the target location. The double hump velocity profile does not occur in either the LAT or FBLAT models suggesting that the lateral connections permitted sensory integration (combining proprioceptive and visual inputs) of the hand positions throughout trials, leading to single humped, smooth velocity profiles. The velocity profiles shown in S6 Fig are single humped in all cases, but similar to Fig 3, there is an abrupt velocity change at timestep 19 for the FF models. In contrast, the LAT model had a smoother velocity profile (compare S6 Fig; third column FF to fourth column LAT).

Similar to VG movements, PPC lateral connections in the MG task also led to smooth velocity profiles (Fig 3C and 3D). In contrast, the FF model’s champion agent only moved towards the target for the 5 timesteps the target was visible (Fig 3C, column 1, timesteps 10–14), emphasizing the purely sensory driven nature of this architecture. Its rates of movement after the visual target extinguished were sporadic and slow, indicating small arbitrary movements generated by the misalignment of the sensory input projections (visual and proprioceptive) across PPC neurons. Similarly, the rates of movement generated by the champion agent for the FB model were primarily driven by the visual representation of the target across the PPC neurons (Fig 3C, column 2). However, the velocity of the LAT and FBLAT models did not decrease upon the disappearance of the visual target at timestep 15 (Fig 3C and 3D, columns 3 and 4), indicating that these models were able to appropriately compensate for an absence of the visual stimulus and the misalignment of sensory inputs.

### 2.6 PPC Evolved Connectivity

**Fig 4:**
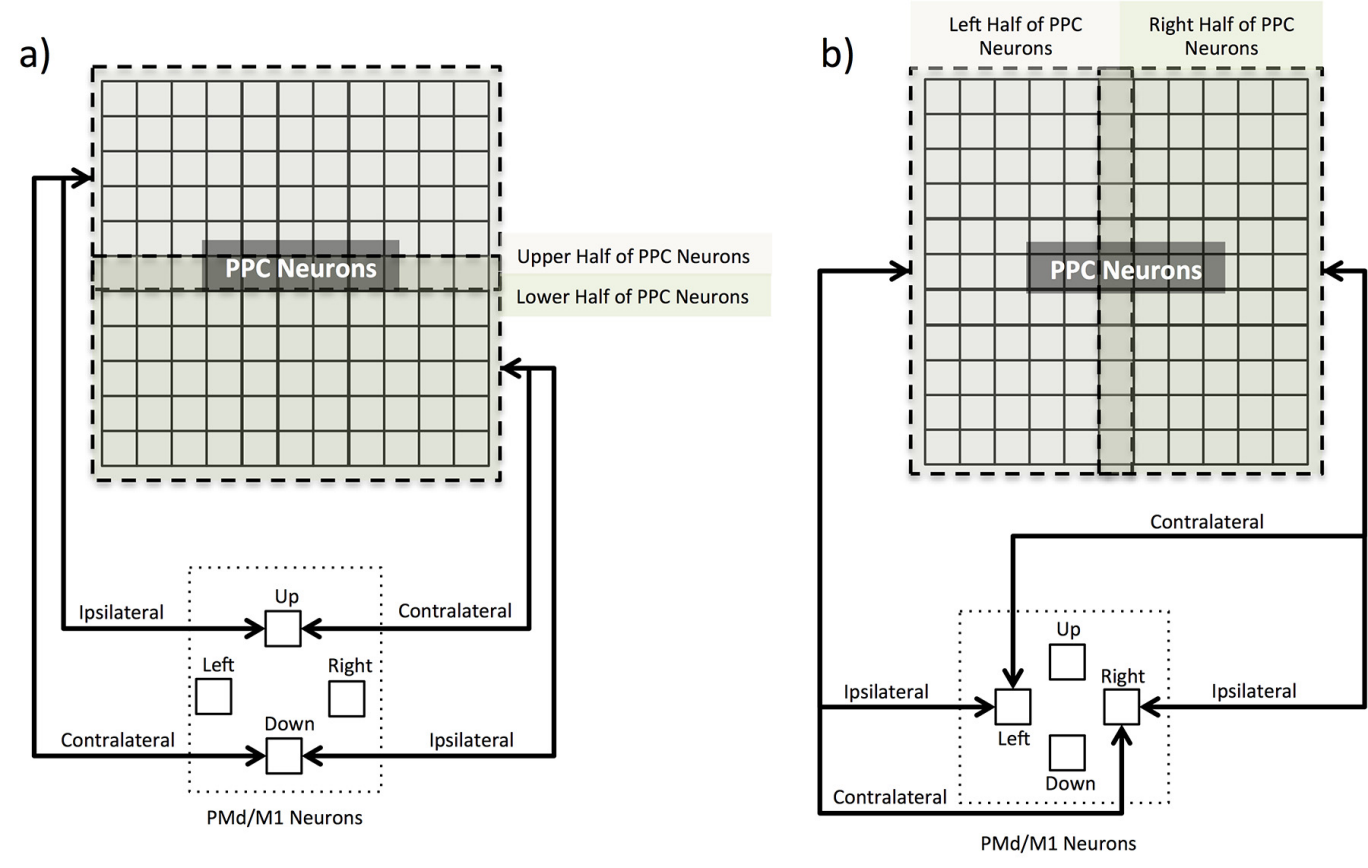
Ipsilateral and Contralateral Connections between PPC and PMd/M1. The images illustrate the difference between ipsilateral and contralateral connections between the PPC and PMd/M1 neurons. The 121 PPC neurons are arranged in a 2-D array (11x11) to illuminate the upper vs. lower and left vs. right halves of PPC neurons connecting to the 4 PMd/M1 neurons. Ipsilateral indicates the half of PPC neurons connected to the corresponding PMd/M1 neuron (e.g., Upper half of PPC neurons to the Up PMd/M1 neuron). Contralateral indicates the half of PPC neurons connected to the opposite PMd/M1 neuron (e.g., Upper half of PPC neurons to the Down PMd/M1 neuron). The ipsilateral and contralateral connections only apply to the feedforward (PPC to PMd/M1 arrows into PMd/M1 neurons) and feedback (PMd/M1 to PPC arrows into PPC neurons) connection sets. a) Ipsilateral and Contralateral connections between the Upper/Lower half of PPC neurons and the Up/Down PMd/M1 neuron. b) Ipsilateral and Contralateral connections between the Left/Right half of PPC neurons and the Left/Right PMd/M1 neuron.

In order to investigate the parameters giving rise to the observed behavior per model, the projections within the PPC (lateral connections), and between the PPC and PMd/M1 (feedforward and feedback connections) were considered. Evolved weights consisted of 3 distinct connection sets (feedforward, feedback, and lateral). All models had 121 PPC neural projections to each of the 4 PMd/M1 neurons (484 feedforward connections). The FB and FBLAT models had 4 PMd/M1 neural projections to the 121 PPC neurons (484 feedback connections) as well. The LAT and FBLAT models had recurrent projections to and from each of the 121 PPC neurons (14641 lateral connections). The feedforward and feedback weights were analyzed with a comparison between the populations averaged ipsilateral vs. contralateral connections (Fig 4). This population level analysis compared the average connection strength between half the PPC neurons and the corresponding PMd/M1 neuron (ipsilateral), to the same half of PPC neurons connected to the opposite PMd/M1 neuron (contralateral). Since the weight matrices had an uneven number of elements (11x11), the middle row (Fig 4A) or column (Fig 4B) was included for both ipsilateral and contralateral projections as a conservative measure.

Recurrent connections within the PPC were analyzed with comparisons of the population averaged connection strength (positive or negative evolved weight value) and distance between the PPC neurons (connection length). Connection length was measured as the Euclidian distance between each PPC neuron determined by their indices (11x11 2-D array of neurons with unique index unit numbering). The PPC connection lengths were binned into short-range (connection length < 5 index units), medium-range (5 index units < = connection length < = 9 index units), and long-range (connection length > 9 index units) projections. The average relative connection strength per binned connection length served as a metric for how the different length connections afforded the emergent behavior in the LAT and FBLAT models.

**Fig 5:**
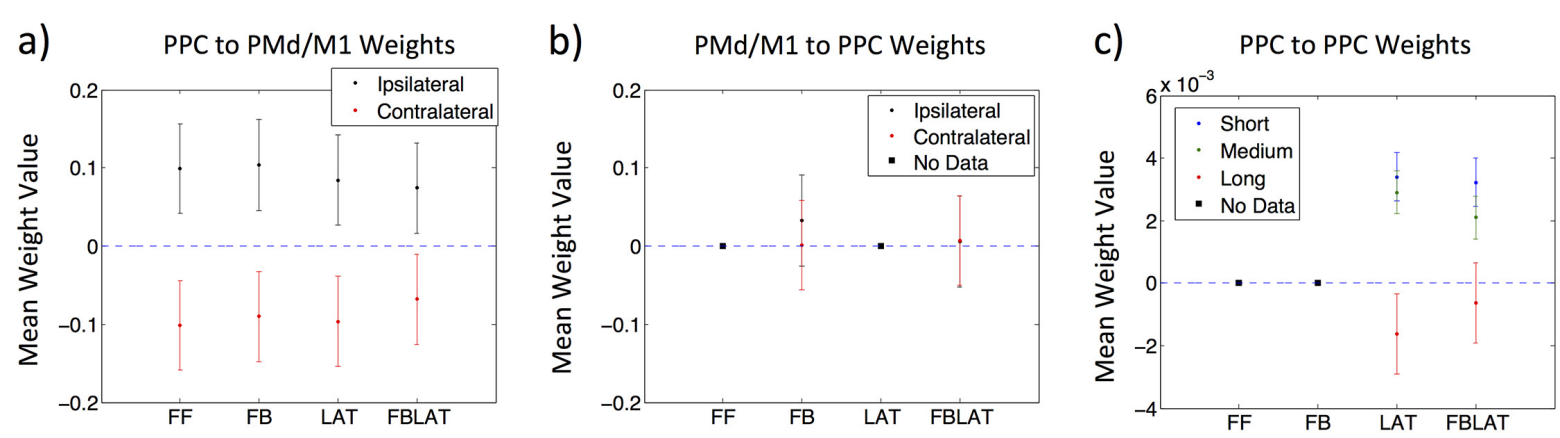
Population Average Connections. The plots show the average weight values per model architecture for feedforward, feedback, and lateral connection sets. In the respective plots, models without the connection set shown are represented by a filled black square (indicated in the Fig. legends) located at a mean weight value of zero. The y-axes show the mean weight value and the x-axes show the model architecture label. The Fig. legends indicate data labels for the respective plots. Error bars show the standard error of the mean (SEM). The dashed line indicates a mean weight value of zero to illuminate differences between excitatory (mean weight value > 0) and inhibitory (mean weight value < 0) weights. a) PPC to PMd/M1 feedforward average ipsilateral vs. contralateral weight values. b) PMd/M1 to PPC feedback average ipsilateral vs. contralateral weight values. c) PPC to PPC lateral average short, medium, and long range weight values.

The evolved feedforward (PPC to PMd/M1) weights in all four architectures (FF, FB, LAT, and FBLAT) showed a distinct pattern of excitatory ipsilateral and inhibitory contralateral projections (Fig 5A). The results suggest that the four models evolved a significant difference (p << 0.001) between excitatory ipsilateral and inhibitory contralateral projections. In contrast to the feedforward weights, models with feedback weights (PMd/M1 to PPC) did not show a significant difference between population-averaged ipsilateral versus contralateral weights (Fig 5B). In fact, the averaged ipsilateral and contralateral weights for both the FB and FBLAT models were not significantly different from zero, indicating that this weight set did not have an impact on the models’ performance in the two tasks. This point is emphasized by the similarities in movement trajectories (Fig 2) and velocity profiles (Fig 3) between FF and FB models, and, LAT and FBLAT models. These results indicate that the FB model behaved like the FF model, and the FBLAT model behaved like the LAT model.

The evolved recurrent connections within PPC tended to be excitatory for local projections and inhibitory for long-range projections (Fig 5C). The LAT model showed a significant difference between population average short-ranged excitatory versus long-ranged inhibitory (p-value << 0.001) and medium-ranged excitatory versus long-ranged inhibitory (p-value << 0.001), but no significant difference between short-ranged excitatory and medium-ranged excitatory lateral weights. Similarly, the FBLAT model showed a significant differences between short-ranged excitatory and long-ranged inhibitory (p-value < 0.01), medium-ranged excitatory and long-ranged inhibitory (p-value < 0.01), but no significant difference between short-ranged excitatory and medium-ranged excitatory weights. These results suggest that the emergence of short/medium-ranged excitatory and long-ranged inhibitory weights allowed models with lateral connections to perform well in the two tasks.

Together, analysis of the connectivity revealed that 1) all four model architectures evolved feedforward weights (PPC to PMd/M1) with ipsilateral excitation and contralateral inhibition, 2) feedback weights (PMd/M1 to PPC) did not have a significant impact on performance, and 3) models with lateral weights (LAT and FBLAT: PPC to PPC) consistently evolved short/medium-ranged excitation and long-ranged inhibition. These results suggest that the observed model behavior emerged from the evolution of ipsilateral excitatory and contralateral inhibitory feedforward weights (all architectures), and short/medium-ranged excitatory and long-ranged inhibitory lateral weights (LAT and FBLAT architectures).

### 2.7 PPC Neural Activity

**Fig 6:**
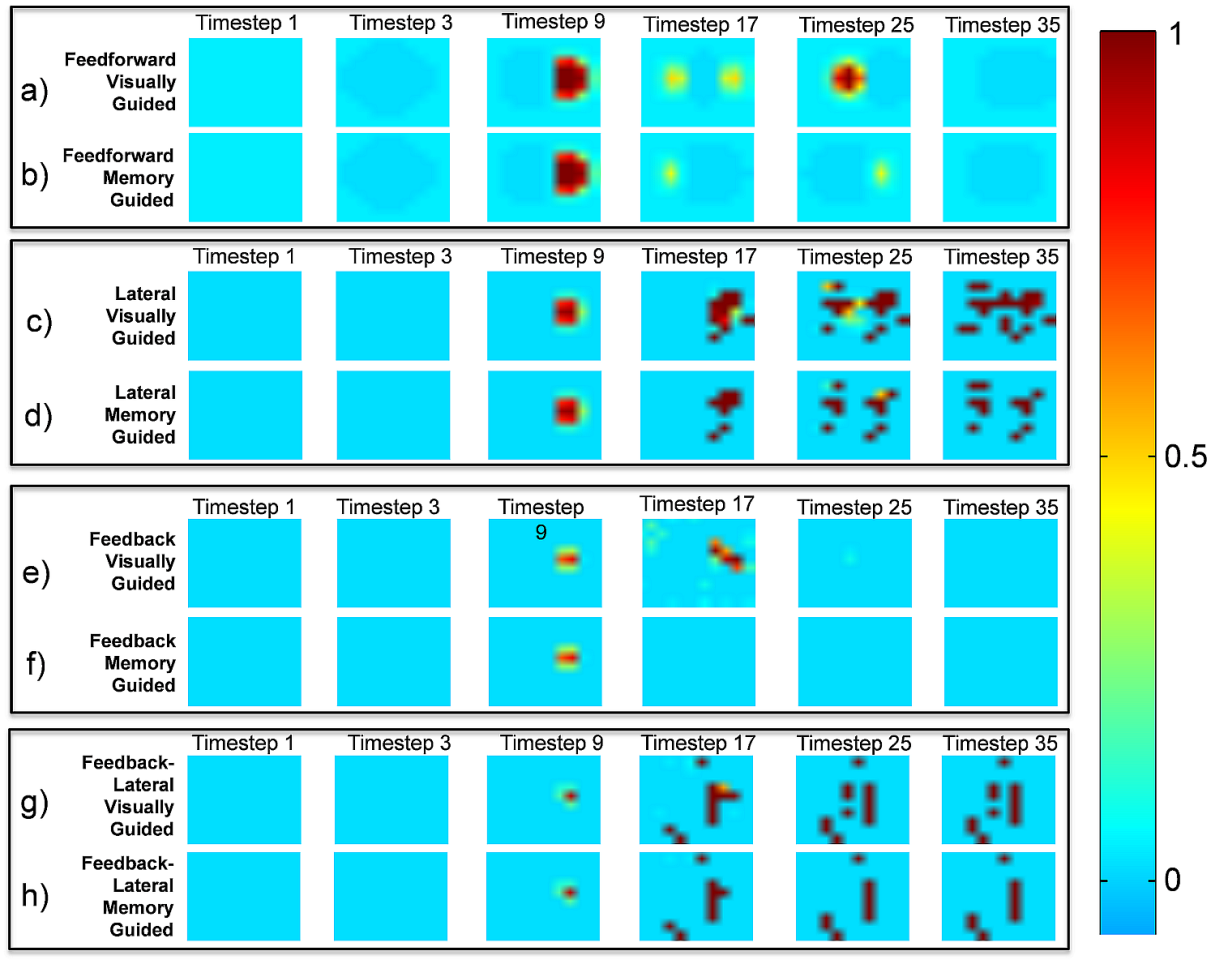
PPC Neural Firing Across a Trial. All plots show a 2-D arrangement of all 121 (11x11) PPC neural firing rates at the timesteps labeled over the columns (from left to right: 1, 3, 9, 17, 25, and 35) for a target located 25 degrees of visual angle to the right of central fixation and the initial hand position. Each pixel is a single PPC neuron’s firing rate at the labeled timestep. The champion agents are shown for the Feedforward, Lateral, Feedback, and Feedback-Lateral models on both the VG and MG tasks. The color bar shows the level of neural activity of the PPC neurons. a) FF model’s champion agent for the VG task. b) FF model’s champion agent for the MG task. c) LAT VG task. d) LAT MG task. e) FB VG task. f) FB MG task. g) FBLAT VG task. h) FBLAT MG task. Compare 6a to 6b and 6c to 6d of the last three columns to see how the models’ PPC neurons fired differently for the two tasks. Compare 6b to 6d (MG task) for timesteps 17, 25, and 35 (columns 4–6) to see how the LAT model was able to reach the target in the absence of a visual stimulus, but the FF model was not.

Evolution of the model architectures allowed the activity of PPC neurons to reflect dynamic motor strategies that integrated visual and proprioceptive inputs to coordinate movement over time. Since the results show that the feedback connections had a minimal effect over model behavior, a comparison between only the FF and LAT models’ champion agents are described, although all four models data are shown for comparison (Fig 6). Fig 6 shows the activity of PPC neurons during a VG and a MG trial for rightward movements (target location = [0°, 25°]). Each colored pixel in Fig 6 represents a single neuron. PPC neural activity is depicted with hues of red, green, and blue to emphasize differences across the trial compared to baseline. For example, a darker hue of blue indicates a decrease in firing rate relative to a neuron’s rate at the beginning of the trial (in the absence of sensory input). Timestep 1 is the start of the trial and shows all neurons’ baseline firing rates. Timestep 3 is when proprioceptive input is first projected onto PPC neurons. Timestep 9 is when visual input is first projected onto PPC neurons. Timestep 17 is during movement. Timestep 25 is when the hand reached the target. Timestep 35 is towards the end of the trial.

In the FF model, the PPC had strong representations of both proprioceptive and visual sensory inputs. Fig 6A shows PPC neural activity for the FF champion during a VG trial. Upon receiving visual input at timestep 9, the PPC activity resulted in the hand moving towards the target (rightward trial). Timestep 17 shows the hand moving at maximum velocity towards the target. Timestep 25 shows the neural activity corresponding to stopping on the target, which occurred when the visual projections (hand and target) overlapped with the proprioceptive projection (hand). The activity representing the proprioceptive location of the hand (dark blue) is at the right hand side as expected, but the activity representing the visual location of the hand is delayed and still near the starting position. Therefore, the (red) activity in the center of neural space is the delayed visual representation of the hand location. PPC activity was suppressed relative to baseline upon timestep 35, when the agent’s hand was static over the target and sensory projections were aligned.

As discussed above, the FF model had difficulties with MG movements. Fig 6B shows PPC neural activity for the FF champion agent during a MG trial. Note the differences between Fig 6A and 6B. When the visual target was no longer available to the agent (Fig 6B, timestep 17), the PPC had no activity associated with the visual target and the agent did not move toward the target location. At timestep 25, visual input from the hand was off center resulting in an inaccurate movement.

In contrast to the FF model, the LAT model made accurate movements in both the VG and MG tasks, which are reflected by the consistent PPC patterns of activity in both tasks (compare Fig 6C to Fig 6D). Unlike the FF model, the LAT model showed less organized neural activity through the trial because sensory input was not the only driving factor. This activity might be a result of an internal motor plan facilitated by intrinsic connectivity, or simply the integration of sensory and lateral inputs. However, the activity resulting from the incorporation of intrinsic inputs allowed the agent to maintain the movement-goal with and without visual sensory input. These additional (intrinsic) inputs may have contributed to the agent’s performance with respect to the inherent sensory delays, as was shown in the movement trajectories and velocity profiles (Figs 2 and 3).

## Discussion

The parietal cortex is needed for integrating sensory information and generating smooth and accurate movements [60]. In the present modeling study, where we used evolutionary algorithms to develop accurate movements, we found that: 1) the lateral connections gave rise to smooth velocity profiles consistent with that observed in visually guided reaching studies, 2) the lateral connections allowed for precise reaching actions in the absence of visual target inputs, 3) the model’s feedforward projections evolved to have ipsilateral excitation and contralateral inhibition, and 4) model’s lateral connections evolved to have short/medium-range excitation and long-range inhibition. We predict that the intrinsic (lateral) connections are necessary for smooth, accurate reaching movements in humans and non-human primates, and lesions or degradation to these connections may result in behavior observed in select sensorimotor deficits such as, optic ataxia or spatial neglect.

Many natural movements (including reaches) show marked acceleration upon movement onset, and rapid deceleration as an end effector nears the task goal (e.g., reaching to a target). This biological movement produces smooth, single humped velocity profiles [54–57]. Interestingly, these profiles only emerged in architectures evolved with intrinsic lateral connections (Fig 3B; LAT and FBLAT). Therefore, the reaching behavior of the LAT and FBLAT models matched subject behavior in control experiments for visually guided reaching. In contrast, jagged or double-humped velocity profiles emerged in the FF and FB models (Fig 3B; FF and FB). This type of velocity profile has been observed in a ‘jumped’ target task [54, 61, 62]. Each hump of the velocity profile indicates a sub-movement towards the motor goal, and the magnitude of a sub-movement is dependent on both, the movement distance and the size of the visual stimulus on the retina [63]. Sub-movements result from a non-optimal trade-off between speed and accuracy, leading to at least two distinct movements (primary movement and corrective movement). Therefore, the evolved lateral connections permitted the emergence of an optimal trade-off between speed and accuracy, eliminating the need for corrective movements.

In support of the hypothesis that intrinsic lateral connections in a brain area like the PPC are needed to produce motor plans, we showed that only the LAT and FBLAT models demonstrated accurate movements in our version of a Memory Guided (MG) reaching task (Fig 2C and S4 and S5 Figs). Since the FB and FBLAT models had reciprocal connections with PMd/M1, and were not able to perform as well as the LAT model in either task, these results indicate that feedback connectivity did not evolve a type of working memory signal that could be used to internally represent the targets. Instead, the evidence suggests that the lateral connectivity evolved to generate a reach-vector to the targets (see also S7 and S8 Figs and S1 Text; PPC Direction Selective Neurons section).

It should be noted that the MG task was not designed to reproduce memory guided tasks such as in Brouwer and Knill [64]. In those experiments there was a movement onset that was delayed for roughly one second after the target was extinguished. In order to simulate these experiments more precisely, working memory areas such as PFC and FEF would need to be added [65]. This is something we plan to explore in future models. However, the present MG task does explore how an area such as PPC can generate an accurate motor plan when visual information about the target location is missing while the movement is being executed.

Similar to the findings from the empirical study by Hwang and Andersen [66], the results shown in Fig 6 (see Fig 6C and 6D) for the LAT model indicate that PPC patterns of activity in the VG task were elevated relative to those in the MG task (compare Fig 6C to Fig 6D). This result suggests that the level of detail represented by the neurons in the simulated PPC could represent local field potentials (LFPs). This activity might be a result of an internal motor plan facilitated by intrinsic connectivity, or simply the integration of sensory and lateral inputs. Either way, the PPC neural activation patterns resulting from the incorporation of intrinsic inputs allowed the agent to maintain the movement-goal with and without visual sensory input, and could be represented by LFPs. Additional empirical studies would need to be performed to elucidate the major contributors towards the movement-goal representation within PPC.

It was somewhat surprising that the feedback connections did not improve performance in the VG and MG tasks. In other studies, models receiving both visual and proprioceptive sensory input that have the ability to perform corrective reaching actions, focus on motor learning from a motor error signal. This has been previously associated with neural activity in the cerebellum [37–39]. The models in the present simulation experiments were not constructed for corrective reaching actions, nor did they have a cerebellum. In contrast to the results in the present study shown for models with feedback connections, empirical studies suggest that feedback connections (from PMd/M1 to PPC) carrying a motor efference copy might result in corrective reaching movements [4, 59, 67–69]. Therefore, we would anticipate that our models with feedback connections (FB and FBLAT) could be evolved to produce corrective reaches in a ‘jumping’ target task. We intend to expand our research in future studies by investigating corrective reaches associated with feedback connections.

Interestingly, transcranial magnetic stimulation experiments (TMS) in MG reaching tasks have shown that a single-pulse over the PPC can cause an increase in pointing scatter [70]. Pointing scatter is comparable to the target error measured in our experiments. This could imply that a TMS pulse over PPC for an MG task renders the lateral connections between PPC neurons inactive, resulting in greater target error, which was similar to the observed behavior of models without lateral connectivity (FF and FB). A future set of simulation experiments could test this prediction by systematically deactivating lateral connections in the LAT and/or FBLAT models and recording the effect over target error in an MG task.

There were at least three strategies for how the different PPC models gave rise to accurate reaching performance: 1) sensory driven through sensory integration alone (FF), 2) sensorimotor integration by combining sensory and feedback (efference copy) inputs (FB and FBLAT), and 3) motor plan (motor-goal or reach-vector) via an internal model by integrating sensory and lateral inputs (LAT and FBLAT). Based on the behavioral, neural, and connectivity analyses, the feedback connections only improved fitness and target error values for the FB model in the VG task, indicating that the motor efference copy was not important for the MG task, and may have had only a minimal effect in the VG task. Evidence for this point comes from the non-significant difference between the evolved average feedback weights (approximately equal to zero) in the FB and FBLAT models. Further evidence is provided by the dramatic difference in performance between the two models with feedback connections in the MG task (Fig 2D; columns 2, 4).

Since the present modeling experiments suggest that the feedback connections did not contribute significantly towards the observed behavior, we consider only the FF and LAT architectures to highlight the difference in sensory driven versus motor plan strategies.

**Fig 7:**
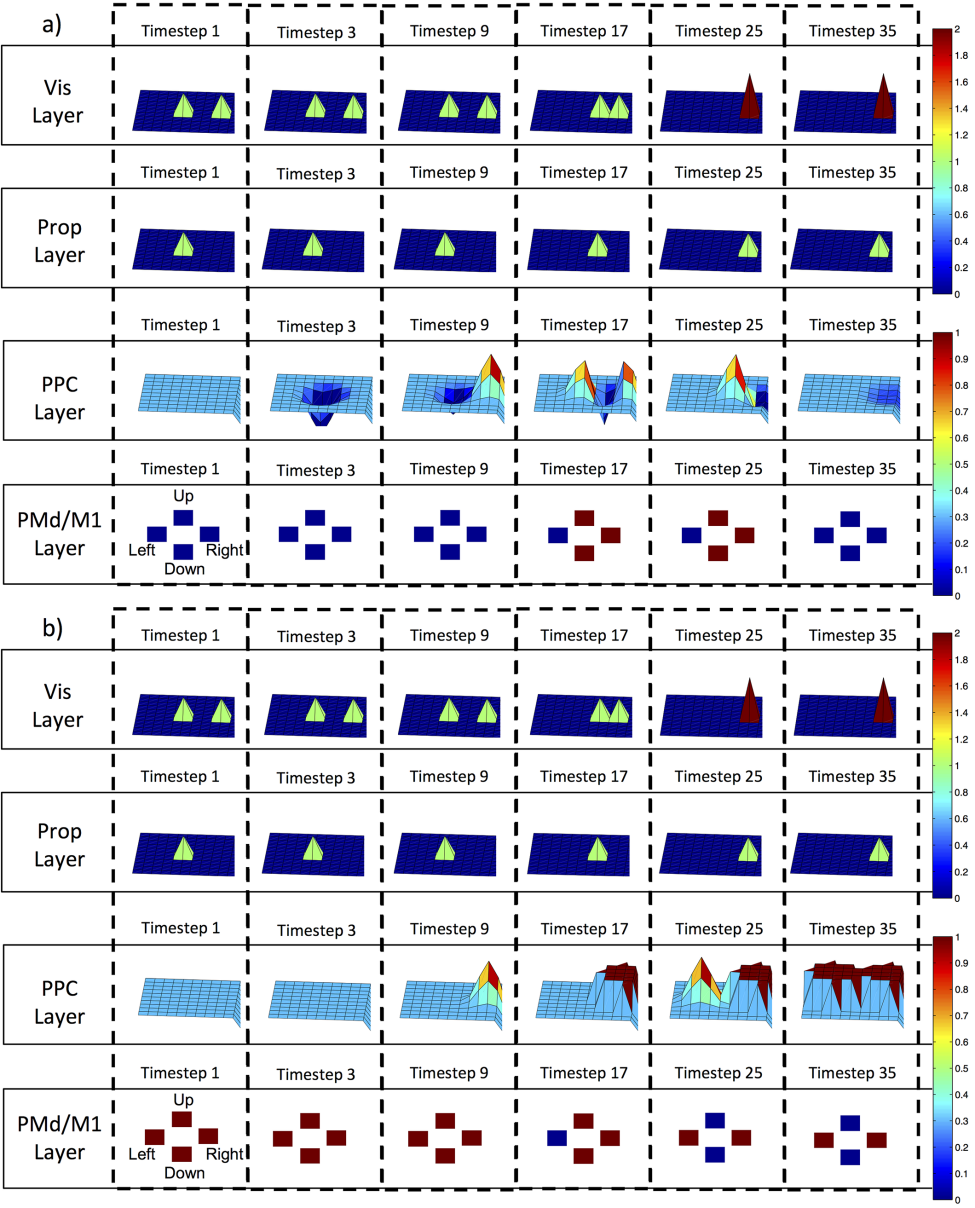
Different strategies taken by the Feedforward and Lateral Models. The colors and corresponding colorbars (right) depict qualitative descriptions of neural activity in the model during a rightward reaching trial in the VG task. The surface plots were derived from the neural activity of the different models and are supplement to the data shown in Fig 6. The Vis Layer (first row) represents the visual input firing rates projected onto the PPC neurons for the corresponding timesteps (columns). The Prop Layer (second row) represents the proprioceptive input firing rates projected onto the PPC neurons. The PPC Layer (third row) represents the PPC neural firing rates that resulted from the sensory inputs with their corresponding delays, and shows how the sensory information was combined during a correct reach to a rightward target. The PMd/M1 Layer shows the four directionally encoded (Up, Down, Left, and Right) PMd/M1 neurons and the respective firing rates for the labeled timesteps. a) Schematic of neural activity for the FF model. The visual input from the Vis Layer at Timestep 1 is reflected in the PPC neural firing rates at Timestep 9. Similarly, the proprioceptive input from the Prop Layer is reflected in the PPC neural firing rates at Timestep 3. The network generated changes in hand position by exciting the PMd/M1 Right neuron. The hand position did not change in the vertical plane (up and down) because both the PMd/M1 Up and Down neurons fired at the same rate. b) Schematic of neural activity for the LAT model. Unlike the FF model, the PPC neurons compensated for the proprioceptive input, which resulted in no change in firing rate across the PPC Layer at Timestep 3. Similarly, the visual representation of the hand location was suppressed as a result of the intrinsic lateral connectivity, which is indicated by the absence of a second bump of activity (small pool of neurons) across the PPC neurons at Timestep 9. The PPC neural activity representing the target location was saturated at Timestep 17, indicated by the dark red hue across a small set of PPC neurons. Changes in the hand position were generated through the suppression of the Left PMd/M1 neuron’s activity while the other three neurons’ firing rates remained saturated. The stopping behavior was generated by the second bump (small pool) of activity across the PPC neurons at Timestep 25, which resulted in a reactivated Left PMd/M1 neuron and complete suppression of both the Up and Down PMd/M1 neurons.

In the sensory driven strategy employed by the FF model, the inputs (Fig 7A, Vis and Prop Layers) directly activated the PPC neurons (Fig 7A, PPC Layer). Upon the first timestep, the activity across the PPC neurons was at a baseline rate (Fig 7A, PPC Layer for Timestep 1). Due to the sensory driven nature of the FF model, the firing rates across the PPC neurons were a linear response from the visual and proprioceptive inputs. The evolved excitatory (ipsilateral) and inhibitory (contralateral) feedforward weights along with the subtractive combination of sensory inputs drove movement in the appropriate direction for the FF model. The PPC activity was a direct response to the visual and proprioceptive activity, and reflected their respective sensory latencies. The activity in the PMd/M1 neurons also reflected the sensory input latencies. Interestingly, there was no PMd/M1 activation before movement, followed by a drive of PMd/M1 activity in the direction of movement, and then no activity when movement stopped (see also S9 Fig and S1 Text; PMd/M1 Neural Strategies section).

In the motor plan strategy, the LAT model was able to overcome sensory latencies and had activity that was not strictly sensory-driven (Fig 7B). The LAT model integrated recurrent lateral and sensory inputs to generate PPC neural activity consistent with empirically derived reach-vector or motor-goal neural activity observed in monkeys [71]. The LAT model compensated for the expected proprioceptive input indicated by the lack of clear proprioceptive representation across the model’s PPC neurons and the fact that the sensory weights were constant across all models. This provides further evidence that an internal representation of the motor-goal must have been encoded across the PPC neural activity. Note how the PPC activity was not a direct consequence of sensory inputs like the FF model. This compensation is indicative of an internal model that encoded a motor-goal, instead of a type of sensory driven memory-like trace.

The data suggest that an internal model emerged as a result of intrinsic excitatory (short/medium-range) and inhibitory (long-range) lateral connections between PPC neurons, which compensated for the sensory consequences of motor actions. The limited sensory influence over the LAT model’s PPC firing rates is illuminated by the large differences in PPC neural activation between FF and LAT models (compare PPC Layer in Fig 7A to Fig 7B). The firing rates across the PPC neurons in the LAT model activated in response to the target location (generating the motor-goal) and another location that corresponded to the effectors initial position (Fig 7B, PPC Layer for Timestep 25). The internal model explanation implies that the motor plan was encoded in the evolved PPC weights before each trial began. This explanation is nicely supported by the consistency in PPC neural activity across the two tasks (VG and MG). The bump of neural activity in the periphery (retino-centric frame) of the PPC neural layer (Fig 7B, PPC Layer for Timesteps 9 and 17) generated movement in the appropriate direction (reach-vector), and then activity in a different location initiated stopping behavior. These two different pools of PPC neurons activated in a sequence that permitted the model to produce smooth and accurate simulated reaching movements. Unlike the sensory-driven strategy evolved in the FF model, movement was initiated by a release of persistent PMd/M1 activity (S9 Fig), causing a drive toward the target, followed by activation in the opposing direction to ‘brake’ movement. Several studies have observed persistent neural activity in VG and MG tasks, similar to that shown for the LAT model (Figs 6C, 6D and 7B), indicating that the simulated neural activation is strongly representative of a biological system. The persistent neural activation has been postulated to represent a directional movement plan [10, 30, 31, 59, 70]. Therefore, our experimental results suggest that the intrinsic lateral connections created an internal model that gave rise to a directional movement plan (motor-goal or reach-vector).

Although evidence for microcircuit inhibition and excitation across PPC neurons is scarce, it was found that cortical columns in frontal cortex exhibit short-range (intracolumnar) excitation and long-range (intercolumnar) or lateral inhibition [72]. Whereas, this microcircuit organization was not tested in PPC, the findings from our study (short/medium-range excitation and long-range inhibition in lateral connections) predict that similar microcircuit organization would be found.

Parieto-frontal neural network models emphasizing biological plausibility in sensorimotor transformation tasks have been used to study the neural underpinnings for competing reaching decisions [35, 36]. In such studies, the models were hardwired, implying that their connectivity was static (hard-coded). In addition, these models only demonstrated unisensory visual input. In contrast, our models demonstrated multisensory (visual and proprioceptive) input with empirically derived delays, and the observed behaviors emerged from evolved connectivity (not hard-coded *a priori*). These differences permitted the model architectures to exhibit multimodal sensory integration and sensorimotor transformations that resulted in smooth, precise reaching actions in the presence and absence of a visual stimulus.

Since the PPC is at the interface between sensory and motor cortices, PPC damage can lead to disorders, which alter the sensorimotor transformations used in hand movements [73]. Optic ataxia (OA) is one such disorder, and can be described as a visually guided reaching disorder attributed to the disconnection between visual information and the motor system [74, 75]. OA results in under-reaching to peripheral visual targets contralateral to the PRR region in the damaged hemisphere [76]. A prediction from our model suggests that damage to the intrinsic lateral connections within a brain region such as PRR would result in behavioral symptoms observed in OA. Additionally, subtypes of spatial neglect have been associated with input-related impairments resulting in slower and lower amplitude initiation and execution of contralateral movements [77]. These observed impairments could also result from specific damage to intrinsic lateral connections within the PPC. It would be of interest to manipulate our models sensorimotor transformations with virtual lesions, for the purpose of investigating disorders such as OA and different subtypes of spatial neglect.

Reference frames are an important component of sensorimotor transformations in the parieto-frontal network, and extensive work has been done to illuminate the specific reference frames contributing to reaching movements [12, 58, 78–83] or a quantitative reach vector. Several studies have suggested that there exists hybrid or intermediate frames of reference across PPC neurons [84–86], and other studies have indicated a primarily gaze-centered frame of reference across PPC neurons [59, 87, 88] in reach related brain areas guiding the calculation and execution of a reach vector. In our study, the reference frame of the PPC neurons was assumed to be gaze centered, though head, eye, and body were aligned. Although we recognize the importance of reference frames in sensorimotor transformations, the present paradigm did not investigate their contributions. However, a future experiment could test reference frame selectivity across the PPC neurons by systematically altering the gaze and hand locations to determine the frames of reference encoded in the simulated neurons when performing visually or memory guided reaches. The feedforward component of the model connecting sensory input layers to the PPC indicated how the PPC neurons received the combined sensory input (i.e., sensory integration), which allowed the PPC to perform a type of vector subtraction similar to what has been shown in empirical studies [58, 59]. The sensory information (visual and proprioceptive positions of hand and target) was combined into a displacement vector defined by extrinsic variables, which guided the evolution of the network connections. Therefore, the eventual reach vector generated by the PPC was agnostic to any prior form of vector calculation. Moreover, the displacement vector information was incomplete in the memory guided task, which was resolved only with the lateral PPC connections.

Taken together, the present set of simulation experiments suggest how intrinsic connections within a cortical region such as parietal cortex can lead to the construction of motor plans. These motor plans can overcome sensory delays and the absence of sensory input while still achieving accurate and rapid movements. Without these intrinsic connections, movements were irregular and prone to errors. This set of experiments revealed the importance of intrinsic PPC lateral connections, and leads to predictions that disruption or destruction of these lateral connections would result in reaching dysfunction similar to that observed in OA or spatial neglect. The results from this study provide theoretical evidence implicating the intrinsic connections within the PPC as an important component in the sensorimotor transformation process. In addition, this theoretical work might motivate neurophysiological studies towards the investigation of intrinsic lateral connections, with intricate experiments involving the blockade or inactivation of these connections while preserving extrinsic connectivity to this region. In summary, this work sheds light on the importance of intrinsic connections within a brain region, and implicates their dynamic recurrent short-range excitatory and long-range inhibitory interactions as the underlying mechanism for which a motor-goal is encoded in the connections leading to biological sensorimotor transformations.

## Materials and Methods

### 4.1 Sensory Inputs

Models received visual sensory inputs from the locations of the hand and the target, in addition to proprioceptive sensory information of the hand location in the reaching space. Each of the sensory network regions (Vision and Proprioception) were a 2-D array (11x11) of neurons, covering the reaching space in degrees of visual angle [-50°, 50°] with 10° of resolution between each neuron. The locations of the hand and the target in reaching space provided sensory information that was transformed into neural activation for corresponding neurons in the 2 different sensory areas (Proprioception and Vision activity set to 1). Neural activation for sensory neurons not corresponding to hand or target locations in the reaching space were set to zero, indicating that neural noise was not present in these simulation experiments. For the Vision area, if the hand and target occupied the same location in reaching space, neural activity for that particular neuron set to 2, indicating a summation of visual sensory inputs.

$$w_{ij}=f\;*\;{cos}^{g}\left(\frac{d}{20}\right)$$ (1)

where *w*_*ij*_ is the weight value or the strength of connection between neuron *j* and neuron *i*, *f* is a constant scalar that took a value of 2 for vision and -4 for proprioception to emphasize a vector subtraction combination of the sensory inputs. The values for the *f* scalar factor were selected to approximately cancel the sensory input signals in PPC neurons as sensory inputs overlapped in the reaching space. The distance *d* indicates the difference between sensory (Vision and Proprioception) and PPC neuron indices (matrix element distances), assuming 2-D retinotopic organization across the sensory neurons. The 2-D cosine peak representing neural activity in PPC neurons resides at the location where a sensory neuron’s index coincides with a PPC neuron’s index (*d* = 0). The gain *g* was set to 200 to ensure that the projections from sensory areas (Proprioception and Vision) to PPC resulted in wide cosine tuning curves activating many PPC neurons (i.e., PPC neurons with large receptive fields).

Derived from empirical work [52], sensory delays were implemented to provide the models with delayed (visual and proprioceptive) information pertaining to hand and target locations in the reaching space. Empirical evidence indicated that proprioceptive inputs have a minimum 30ms delay and visual inputs have a minimum 90ms delay before these sensory signals activate PPC neurons. To implement these minimum biological latencies, PPC neurons received proprioceptive information of the hand’s location in the reaching space delayed by 3 timesteps and visual input (of hand and target) delayed by 9 timesteps. This indicates that our models’ PPC neurons did not acquire visual sensory input until the 9^th^ timestep of each trial, implying that the models were blind to the goal of the task for the first 1/5^th^ of every trial. Proprioceptive input was acquired upon the 3^rd^ timestep of every trial, however, it provided no relevant information pertaining to the motor-goal of the task (i.e., the location of the target in reaching space).

### 4.2 PPC and PMd/M1 Neural Firing

PPC and PMd/M1 neural activation was generated by a sigmoid function shown in Eq 2 that inherently bounds values between [0, 1].

$$s_{i}(t)=\frac{1}{1+{exp}^{(\Delta -I_{i}(t))*k}}$$ (2)

*s*_*i*_ *(t)* is a firing rate for PPC or PMd/M1 neuron *i* at timestep *t*, *Δ* is the evolved bias value on the sigmoid that controls the range of values a neuron can respond to (activation range) *I*_*i*_ *(t)* shown in Eq 3 is the synaptic input (from all connected neurons) to neuron *i* at timestep *t*, and *k* is the evolved gain for the sigmoid that determines the amount of input values a neuron can respond to (neural sensitivity). Neurons in the same layer (PPC or PMd/M1) shared identical bias and gain values, however, neurons in different layers could have different values. The synaptic input *I*_*i*_ *(t)* is shown in Eq 3:

$$I_{i}(t)=\sum_{j}w_{ij}s_{j}(t-1)$$ (3)

where *I*_*i*_ *(t)* is the total input to neuron *i* at timestep *t*, *w*_*ij*_ is the weight or strength of connection from neurons *j* to *i*, and *s*_*j*_ *(t-1)* is the previous firing rate for neuron *j*.

PPC and PMd/M1 bias and gain parameters were evolved using an evolutionary algorithm, resulting in a total of four neural firing parameters evolved for each model (two for each the PPC and PMd/M1 neural layers). The values for *Δ* (bias) and *k* (gain) were continuous and in the ranges [-5, 5] and [0.1, 10] respectively.

### 4.3 Movements

Population vector coding was used to determine the rate and direction of hand movements. The activity of each PMd/M1 neuron was multiplied by the sine and cosine of their respective angles in polar coordinates (Up: π/2, Left: π, Down: 3π/2, and Right: 2π), resulting in a vector sum that provided the vertical and horizontal components of hand movements. This method is called population coding [89] and is shown in Eq 4.

$$\begin{array}{l} D_{i}\;*\;\sum_{j}\sin(\theta_{j}-\varphi_{i})\;*\;s_{j}(t)\;\left\{ \begin{array}{l} i=1,2\\ j=1,\ldots ,4 \end{array}\right.\\ \theta_{j}=(j-1)\;*\;\frac{\pi }{2}\\ \varphi_{i}=(i-1)\;*\;\frac{\pi }{2} \end{array}$$ (4)

*D*_*i*_ is the population vector with vertical (*i = 1*) and horizontal (*i = 2*) components, the index *j* represents the four directionally encoded PMd/M1 neurons, *θ*_*j*_ represents the encoded angles corresponding to right (0°), up (90°), left (180°), and down (270°) respectively, *φ*_*i*_ changes the angle *θ* by pi/2 effectively taking the cosine for the horizontal component of the population vector, *s*_*j*_ *(t)* is the neural activation for neuron *j* at timestep *t*, and *v* is a constant set to 2 for the maximum hand velocity at 2 degrees/timestep (i.e., 200 degrees/second) while moving to horizontal and vertical targets (e.g., D_1_ = 2.0 and D_2_ = 0), and 2.83 degrees/timestep (283 degrees/second) for moving to diagonal targets (e.g., D_1_ = 2.0 and D_2_ = 2.0). This bound on movement rate was selected to match biological limitations recorded from empirical reaching studies [54, 57].

### 4.4 Evolutionary Algorithm

The neural networks were tuned over 25000 generations utilizing evolutionary computation from an open source library (Evolving Objects- http://eodev.sourceforge.net) [90]. After 25000 generations of parameter tuning, the best agents were selected based on minimized fitness (lower fitness was better) (see S1 Text; Fitness Calculations section). The evolved free parameter labels and their respective value ranges were: 1) the amount of connection weights depending on model architecture (FF: 484; FB: 968; LAT: 15125; FBLAT: 15609) in the range of [-1.0, 1.0], 2 sigmoid bias parameters [-5.0, 5.0] (see Δ in Eq 2) and 2 sigmoid gain parameters [0.1, 10] (see k in Eq 2), 1 gain and 1 bias parameter for each the PMd/M1 and PPC neural layers. All parameters were continuous floats resulting in an extremely large search space. The EA used in our experiments is the evolution strategy algorithm “mu comma lambda” (μ, λ), which included elitism, crossover with roulette wheel selection, and mutation [53]. Each generation contained 20 agents with a 0.4 probability of receiving mutations. If an agent was selected for mutation, a random number drawn from a Gaussian distribution (Gaussian distribution was determined by upper and lower bounds from parameter range) was added randomly (50% chance) to the set of free parameters. The 3 different Gaussian distributions were: 1) G(0,0.3) for the weights, 2) G(0, 3) for the biases, and 3) G(0, 1.5) for the gains. 100 evolution strategy algorithms were run (per model), each with different seeds to establish the 100 independently evolved best agents (one best agent per EA).

The fitness function shown in Eq 5 was utilized to quantify each agent’s speed and precision throughout the 8 trials. The fitness function captured their ability to remain at the target locations without over/under-shooting or jittering (small undesired movements). An agent’s fitness was the Euclidian distance between the hand and target locations summed over every timestep and trial.

$$\begin{array}{l} F=\sum_{n}\sum_{t}E_{n}(t)\;\left\{ \begin{array}{l} n=1,\ldots ,8\\ t=1,\ldots ,50 \end{array}\right.\\ E_{n}(t)=\sqrt{{\left(H_{x}(t)-T_{xn}\right)}^{2}+{\left(H_{y}(t)-T_{yn}\right)}^{2}} \end{array}$$ (5)

*F* is the fitness value, *n* is the trial number from 1 to 8, *t* is the timestep from 1 to 50, *E*_*n*_ *(t)* is Euclidian distance between the hand and target locations for timestep *t* on trial *n*, (*H*_*x*_ *(t)*, *H*_*y*_ *(t))* are the horizontal and vertical hand position components in 2-D Cartesian reaching space, and (*T*_*xn*_, *T*_*yn*_ *)* is the coordinate for target position on trial *n*.

The minimum (best possible fitness value) fitness an agent could achieve was 3563.4, corresponding to the summed Euclidian distance across all timesteps and trials when an agent performed perfectly (moved at maximum rate with no deviation in path and stopped exactly on the target for every trial). To adjust for this, 3563.4 was subtracted from the agents’ fitness values to normalize fitness indicating optimization at 0 (S2 Fig).

## Supporting Information

S1 Dataset Raw Data. The complete dataset utilized in the data-dependent figures.
(ZIP)

S1 Fig Reaching Tasks. The two tasks (Visually & Memory Guided Reach) are organized identically with eight potential peripheral target locations either 25° (vertical and horizontal targets) or 35° (diagonal targets) of visual angle away from the central position. Every trial for both tasks starts out with the fixation and hand aligned at the central position. For both tasks, one of the target locations is illuminated (provides visual input to the models) at the start of a trial, then for the visually guided task the target stays illuminated for the remainder of the trial, while the target disappears after 50ms (5 timesteps) for the memory guided task. The goal in each task is to keep fixation centered while moving the hand to the target location as quickly as possible and holding the hand at the target location for the remainder of the trial.
(TIF)

S2 Fig Evolution of Fitness. Each plot shows the evolution of the fitness values for the best agents of 100 independent evolutionary algorithms (EAs) (supplement to Fig 2). Fitness values were minimized and corrected, which indicates that the best possible fitness value was 0. The black line is the mean fitness of all 100 agents with the width of the line showing the standard deviation around the mean at every generation. The red dashed line is the evolution of fitness for the champion agent at the last generation. The y-axes show the corrected fitness values calculated from the summed Euclidian distance in degrees of visual angle between the hand and the target for every timestep across all trials. The x-axes show the generation number, going from 1 to 25000. a) Evolution of fitness values for the FF model. b) Evolution of fitness values for the FB model. c) Evolution of fitness values for the LAT model. d) Evolution of fitness values for the FBLAT model.
(TIF)

S3 Fig Population Fitness. The fitness values of the 100 agents after 25000 generations of evolution is supplemental to Fig 3. Each data point (black dot) represents a single agent’s fitness value. The y-axes show the fitness values calculated for the tasks, with the scales set (VG: 0–2500; MG; 0–6000) to illuminate the differences between the models. The x-axes show the different models (FF: Feedforward, FB: Feedback, LAT: Lateral, FBLAT: Feedback-Lateral). a) Population fitness values for the 100 best agents in the visually guided (VG) task. b) Population fitness values for the 100 best agents in the memory guided (MG) task.
(TIF)

S4 Fig Fitness and Target Error: VG and MG Tasks. The fitness values are the summed Euclidian distance from the target at every timestep across all 8 trials. The fitness values were corrected by subtracting off the minimum possible fitness value for each trial to make the best possible fitness value equal to 0. The target error, which is given in degrees of visual angle, is the average Euclidian distance and standard deviation from the target across the trials, with the minimum target error equal to 0. The columns show the data from the four models (from left to right: FF: Feedforward, FB: Feedback, LAT: Lateral, FBLAT: Feedback-Lateral). The champion agent had the best fitness of the 100 independently evolved agents per model. The top four rows (rows 1–4) represent data from the VG task and the last four rows (rows 5–8) represent data from the MG task (supplement to Fig 2).
(TIF)

S5 Fig p-Value Comparisons. All data shown, reflect p-values calculated with Wilcoxon rank sum tests of pair wise comparisons between medians of data from the models labeled in the 1^st^ column (FF: Feedforward model; FB: Feedback model; LAT: Lateral model; FBLAT: Feedback-Lateral model). Bold values indicate significance (α = 0.05, p < 0.008 Bonferroni corrected for multiple comparisons). The labeled columns (Fitness Medians; Population TE Medians; Champion TE Medians) for the 2 tasks (Visually Guided and Memory Guided) indicate the pair wise comparison between the medians from the distribution of fitness values for all 100 agents per model, the medians from the distribution of target error (TE) for the population of 100 agents per model, and the medians from the distribution of TE for only the champion agents per model (supplement to Fig 3).
(TIF)

S6 Fig Reaching Trajectories and Velocity Profiles with Different Sensory Weights (FF and LAT Models). The data shown are comparable to that shown in Figs 2 and 3 for the Feedforward (FF) and Lateral (LAT) models. The difference, is the sensory input weight scale factor (*f*) is set to +4 for Vision and+4 for Proprioception (see Eq 1; *f* parameter) to contrast the subtractive weight data shown in Figs 2 and 3 (Eq 1; *f* set to +2 for Vision and -4 for Proprioception). The first two columns show reaching trajectories for the FF (column 1) and LAT (column 2) models similar to Fig 2. The third and fourth columns show the average velocity profile for the FF and LAT models respectively, similar to Fig 3. a) The champion agents’ reaching trajectories (FF: column 1; LAT: column 2) and velocity profiles (FF: column 3; LAT: column 4) for the VG task. b) The means and standard error of the means (SEMs) for reaching trajectories (FF: column 1; LAT: column 2) and velocity profiles (FF: column 3; LAT: column 4) for the VG task. c) The champion agents’ reaching trajectories (FF: column 1; LAT: column 2) and velocity profiles (FF: column 3; LAT: column 4) for the MG task. d) The population means and SEMs for reaching trajectories (FF: column 1; LAT: column 2) and velocity profiles (FF: column 3; LAT: column 4) for the MG task.
(TIF)

S7 Fig PPC Neural Direction Selectivity. Representative directionally selective PPC neurons that supplement the data shown in Fig 4. The polar angle plots depict the normalized firing rate of different PPC neurons corresponding to different directions of movement. The central plots depict exemplars of non-directionally selective neurons. a) FF. b) FB. c) LAT. d) FBLAT.
(TIF)

S8 Fig Direction Selectivity Index. The top plots of a-d show the direction selectivity index for all PPC neurons that fired at a rate > = 0.1 at any timestep during a trial. The text at the bottom of the plots indicates the number of PPC neurons that did not meet the criteria. The y-axis shows the magnitude of direction selectivity and the x-axis shows the angle of direction selectivity. The black vertical dashed lines represent the directions of the targets during the trials. a) FF. b) FB. c) LAT. d) FBLAT. The plots in a-d show that all model types evolved strong directionally selective PPC neurons for each of the trials (also see S5 and S7 Figs).
(TIF)

S9 Fig PMd/M1 Neural Strategies for the Top 25 Fittest Agents. The schematic drawings in a-g represent the premotor/primary motor neural strategies for generating reaching trajectories to the correct targets across all trials for the 25 fittest agents of each model in the VG task. The y-axes of a-g represent the firing rate of opposing pairs of PMd/M1 neurons, which give rise to movement in all trials. The x-axes of a-g show the temporal progression of a trial (50 timesteps approximating 500ms). Neural strategy schematics a-g, are broken into three phases in temporal order; trial onset phase, movement onset phase, and movement offset phase. These three phases account for the PMd/M1 neural firing across the duration of the trial. The blue line represents the neural activity for the direction of hand movement (e.g., the Right neuron). The red line represents the neural activity for the direction counter to the hand’s trajectory towards the target (e.g., the Left neuron). The solid black lines represent an overlap of activity between the blue and red lines. With the exception of b, c, and e, all strategies either had initial activity near the minimum or maximum. In neural strategies b, d, and e the initial activity was in the range of [0.3, 0.9]. Plots h-k show histograms of the distributions of neural strategies used by the top 25 fittest agents per model. The combined strategies (a+g, d+e, and c+f) indicate that some agents had opposing pairs of neurons firing differently on all trials (e.g., the Up and Down neurons used a different strategy than the Right and Left neurons). The label ‘inconclusive’ in h-k, represent agents that did not perform well on all the trials yielding incorrect trajectories as a result of poorly defined neural strategies. a-g) PMd/M1 neural strategies for generating movements. h) Histogram of the Feedforward model’s top 25 agents’ neural strategies. i) Histogram of the Feedback model’s top 25 agents’ neural strategies. j) Histogram of the Lateral model’s top 25 agents’ neural strategies. k) Histogram of the Feedforward model’s top 25 agents’ neural strategies. The plots in h-k show that the more complex models evolved a more diverse strategy distribution (compare j and k to h and i).
(TIF)

S1 Text Supplementary Text. Evolutionary Algorithm section—details about the evolutionary algorithm utilized to tune free parameters in the simulation experiments; Fitness Comparisons section—comparisons of the models’ parameter evolution pertaining to the emergent behavior; Target Error Comparisons section—statistical comparisons of model accuracy in the two simulated reaching tasks (VG and MG); PPC Direction Selective Neurons section—description of the analysis and results showing how individual PPC neurons developed preferred direction selectivity, indicating their neural activation for only select trials; PMd/M1 Neural Strategies section—description of the procedure that distinguished between the different types of neural patterns in the output (PMd/M1) layer that led to good fitness values; Supplemental References section—list of references only cited in S1 Text sections.
(DOCX)
