# Supplementary figures and images for: The Importance of Lateral Connections in the Parietal Cortex for Generating Motor Plans
Source: PLoS One. 2015 Aug 7;10(8):e0134669. doi: 10.1371/journal.pone.0134669 (PMC4529220; doi:10.1371/journal.pone.0134669)

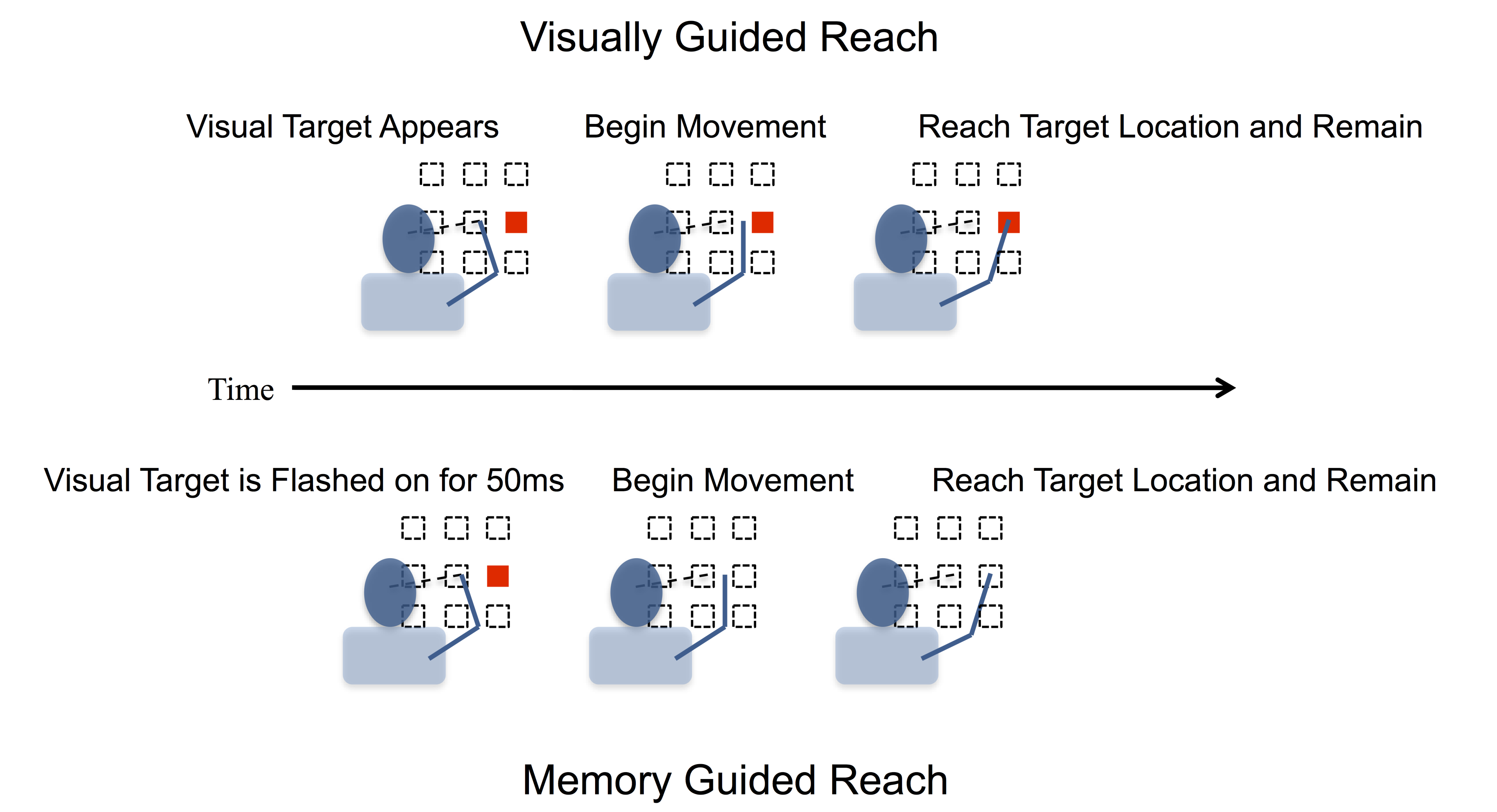

Supplement: S1 Fig — The two tasks (Visually & Memory Guided Reach) are organized identically with eight potential peripheral target locations either 25° (vertical and horizontal targets) or 35° (diagonal targets) of visual angle away from the central position. Every trial for both tasks starts out with the fixation and hand aligned at the central position. For both tasks, one of the target locations is illuminated (provides visual input to the models) at the start of a trial, then for the visually guided task the target stays illuminated for the remainder of the trial, while the target disappears after 50ms (5 timesteps) for the memory guided task. The goal in each task is to keep fixation centered while moving the hand to the target location as quickly as possible and holding the hand at the target location for the remainder of the trial. (TIF) [file pone.0134669.s002.tif]

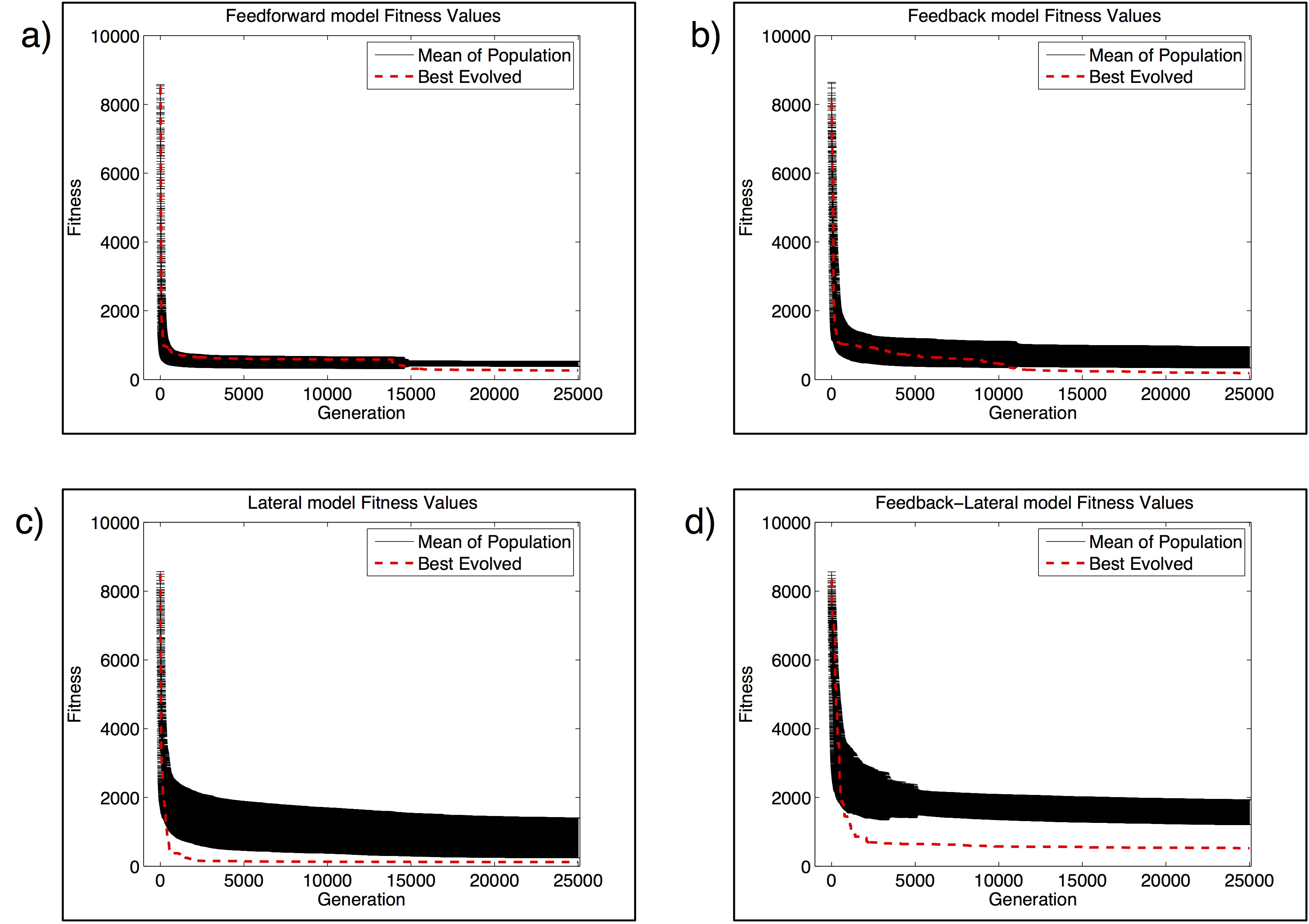

Supplement: S2 Fig — Each plot shows the evolution of the fitness values for the best agents of 100 independent evolutionary algorithms (EAs) (supplement to Fig 2). Fitness values were minimized and corrected, which indicates that the best possible fitness value was 0. The black line is the mean fitness of all 100 agents with the width of the line showing the standard deviation around the mean at every generation. The red dashed line is the evolution of fitness for the champion agent at the last generation. The y-axes show the corrected fitness values calculated from the summed Euclidian distance in degrees of visual angle between the hand and the target for every timestep across all trials. The x-axes show the generation number, going from 1 to 25000. a) Evolution of fitness values for the FF model. b) Evolution of fitness values for the FB model. c) Evolution of fitness values for the LAT model. d) Evolution of fitness values for the FBLAT model. (TIF) [file pone.0134669.s003.tif]

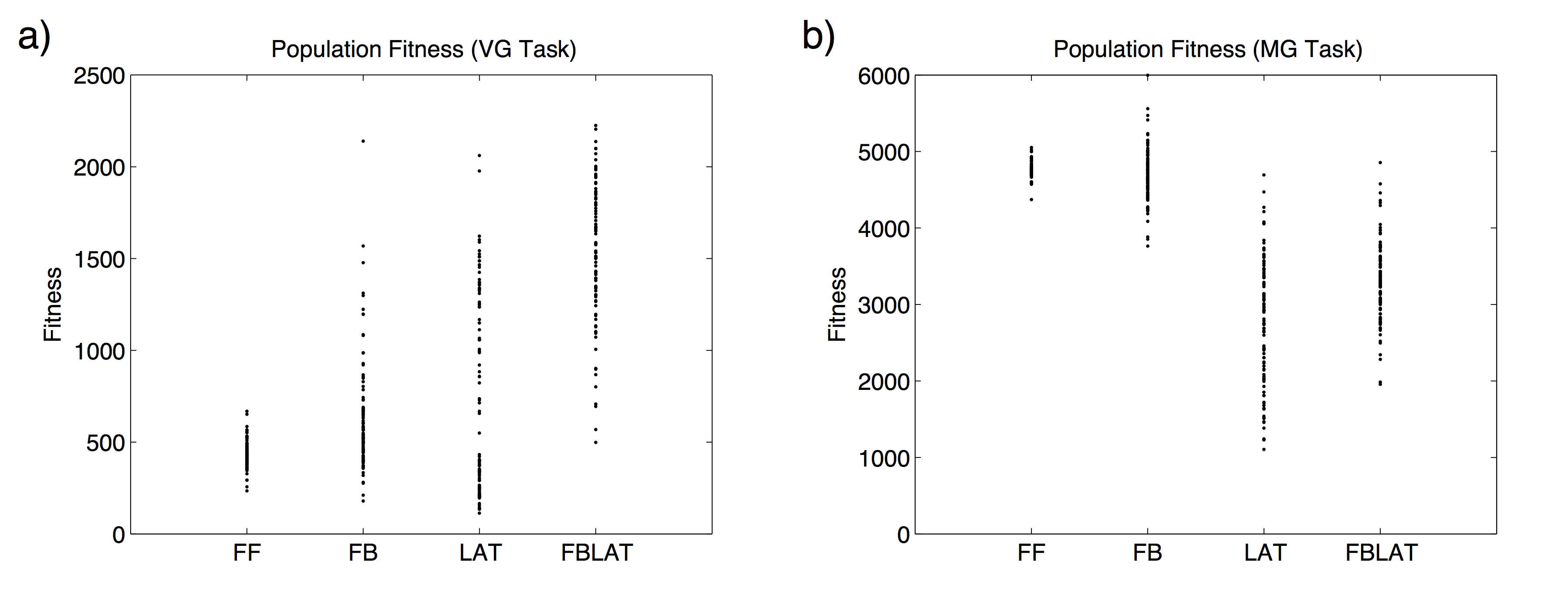

Supplement: S3 Fig — The fitness values of the 100 agents after 25000 generations of evolution is supplemental to Fig 3. Each data point (black dot) represents a single agent’s fitness value. The y-axes show the fitness values calculated for the tasks, with the scales set (VG: 0–2500; MG; 0–6000) to illuminate the differences between the models. The x-axes show the different models (FF: Feedforward, FB: Feedback, LAT: Lateral, FBLAT: Feedback-Lateral). a) Population fitness values for the 100 best agents in the visually guided (VG) task. b) Population fitness values for the 100 best agents in the memory guided (MG) task. (TIF) [file pone.0134669.s004.tif]

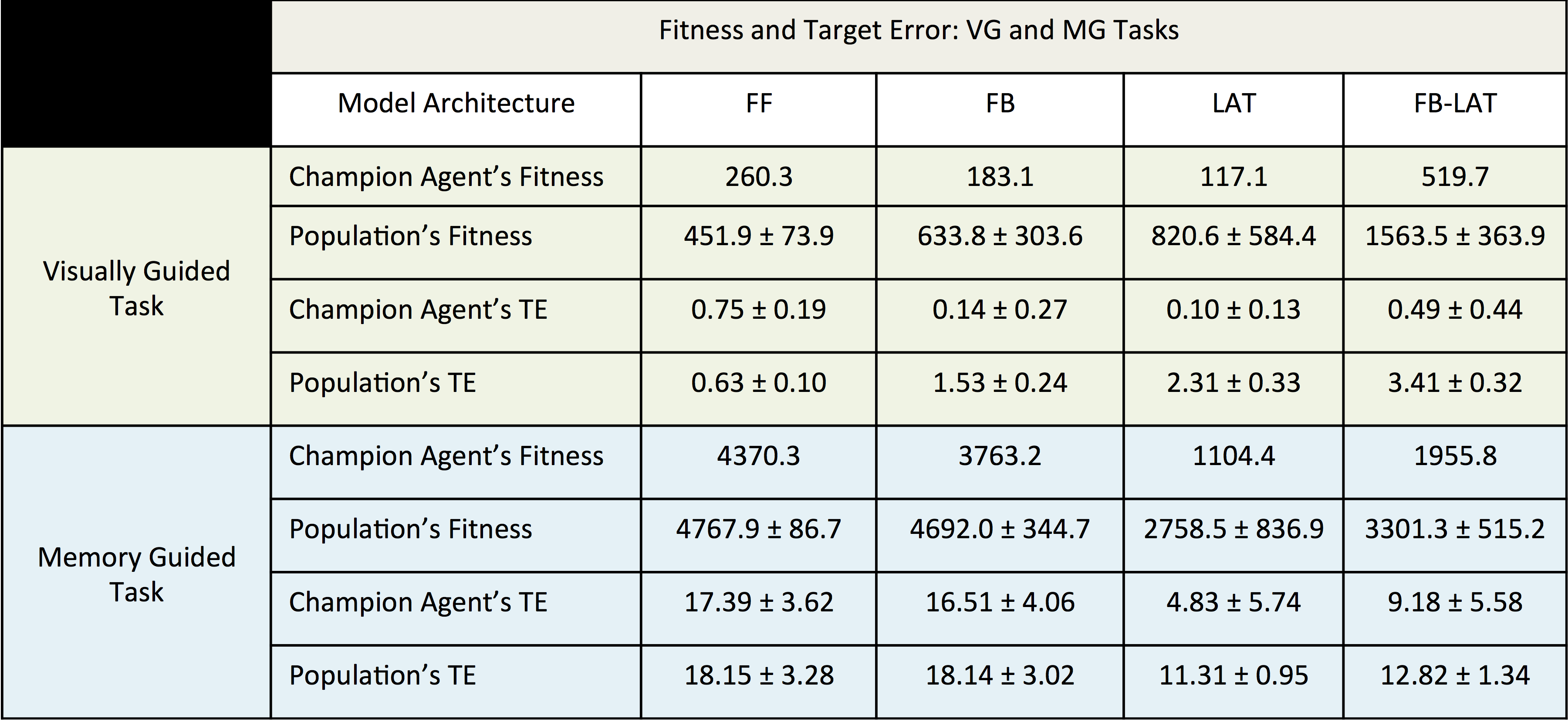

Supplement: S4 Fig — The fitness values are the summed Euclidian distance from the target at every timestep across all 8 trials. The fitness values were corrected by subtracting off the minimum possible fitness value for each trial to make the best possible fitness value equal to 0. The target error, which is given in degrees of visual angle, is the average Euclidian distance and standard deviation from the target across the trials, with the minimum target error equal to 0. The columns show the data from the four models (from left to right: FF: Feedforward, FB: Feedback, LAT: Lateral, FBLAT: Feedback-Lateral). The champion agent had the best fitness of the 100 independently evolved agents per model. The top four rows (rows 1–4) represent data from the VG task and the last four rows (rows 5–8) represent data from the MG task (supplement to Fig 2). (TIF) [file pone.0134669.s005.tif]

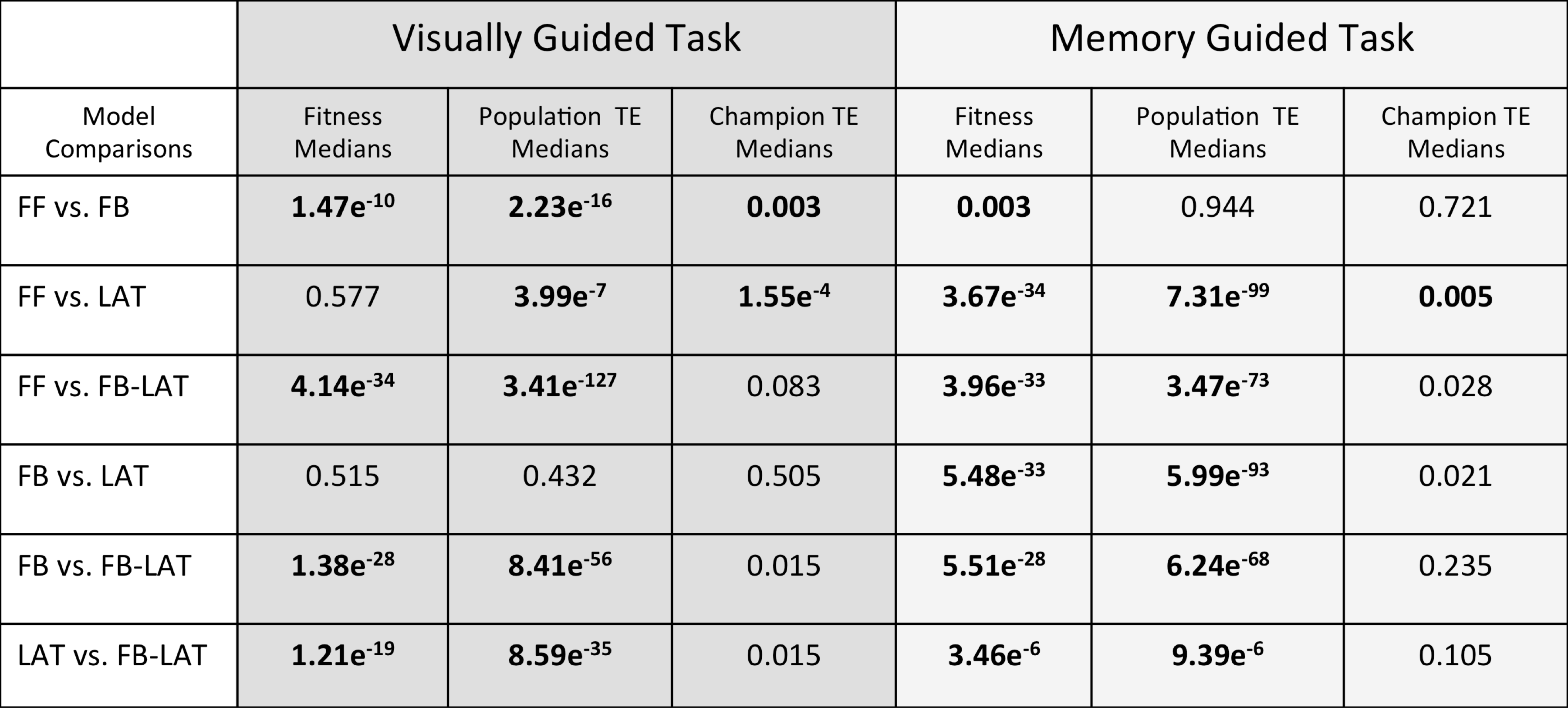

Supplement: S5 Fig — All data shown, reflect p-values calculated with Wilcoxon rank sum tests of pair wise comparisons between medians of data from the models labeled in the 1st column (FF: Feedforward model; FB: Feedback model; LAT: Lateral model; FBLAT: Feedback-Lateral model). Bold values indicate significance (α = 0.05, p < 0.008 Bonferroni corrected for multiple comparisons). The labeled columns (Fitness Medians; Population TE Medians; Champion TE Medians) for the 2 tasks (Visually Guided and Memory Guided) indicate the pair wise comparison between the medians from the distribution of fitness values for all 100 agents per model, the medians from the distribution of target error (TE) for the population of 100 agents per model, and the medians from the distribution of TE for only the champion agents per model (supplement to Fig 3). (TIF) [file pone.0134669.s006.tif]

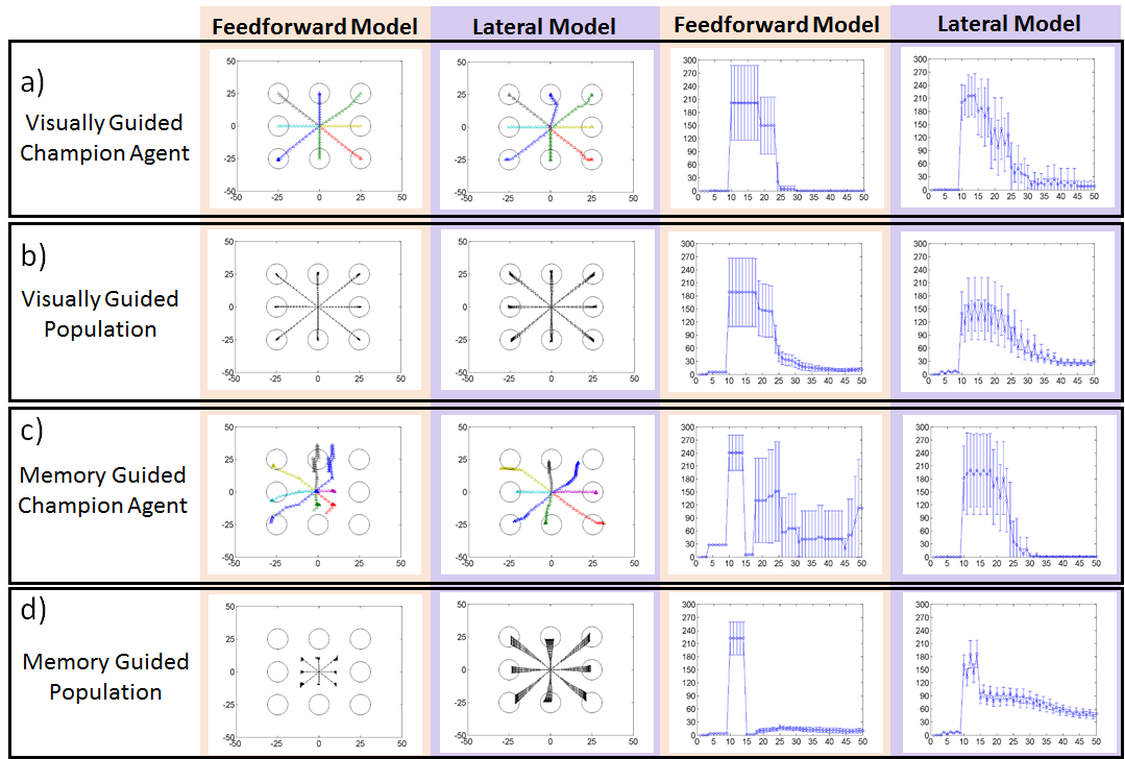

Supplement: S6 Fig — The data shown are comparable to that shown in Figs 2 and 3 for the Feedforward (FF) and Lateral (LAT) models. The difference, is the sensory input weight scale factor (f) is set to +4 for Vision and+4 for Proprioception (see Eq 1; f parameter) to contrast the subtractive weight data shown in Figs 2 and 3 (Eq 1; f set to +2 for Vision and -4 for Proprioception). The first two columns show reaching trajectories for the FF (column 1) and LAT (column 2) models similar to Fig 2. The third and fourth columns show the average velocity profile for the FF and LAT models respectively, similar to Fig 3. a) The champion agents’ reaching trajectories (FF: column 1; LAT: column 2) and velocity profiles (FF: column 3; LAT: column 4) for the VG task. b) The means and standard error of the means (SEMs) for reaching trajectories (FF: column 1; LAT: column 2) and velocity profiles (FF: column 3; LAT: column 4) for the VG task. c) The champion agents’ reaching trajectories (FF: column 1; LAT: column 2) and velocity profiles (FF: column 3; LAT: column 4) for the MG task. d) The population means and SEMs for reaching trajectories (FF: column 1; LAT: column 2) and velocity profiles (FF: column 3; LAT: column 4) for the MG task. (TIF) [file pone.0134669.s007.tif]

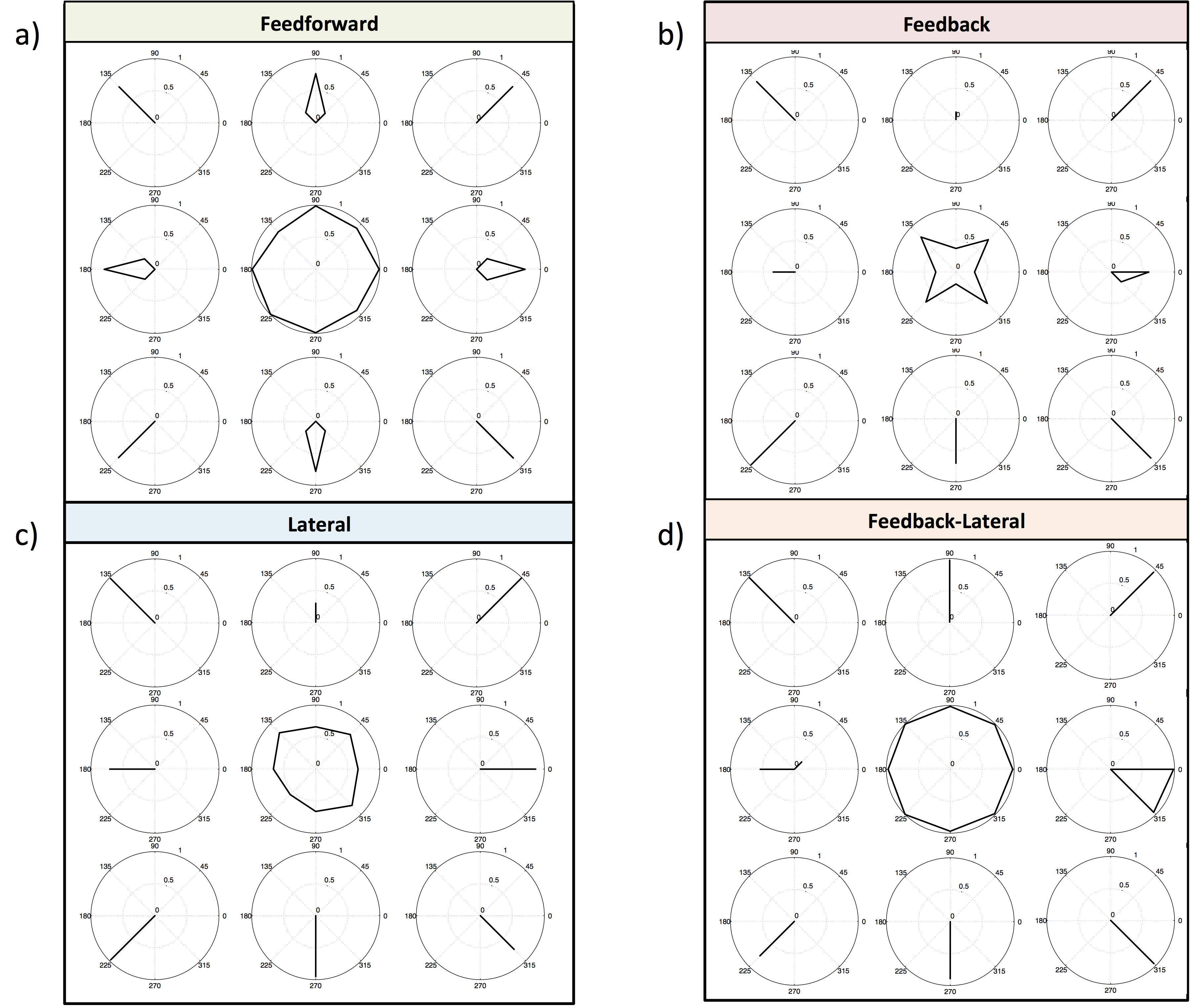

Supplement: S7 Fig — Representative directionally selective PPC neurons that supplement the data shown in Fig 4. The polar angle plots depict the normalized firing rate of different PPC neurons corresponding to different directions of movement. The central plots depict exemplars of non-directionally selective neurons. a) FF. b) FB. c) LAT. d) FBLAT. (TIF) [file pone.0134669.s008.tif]

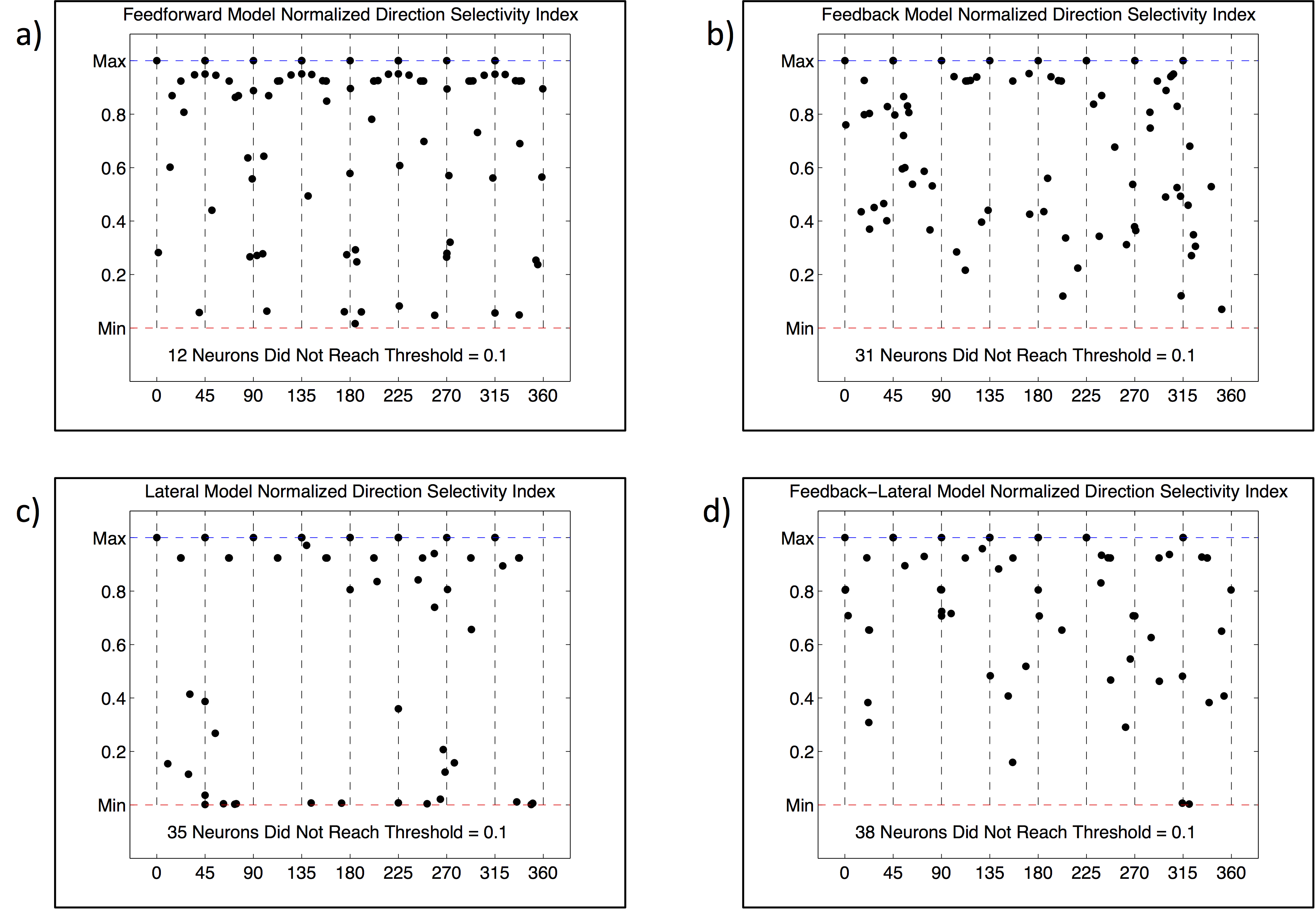

Supplement: S8 Fig — The top plots of a-d show the direction selectivity index for all PPC neurons that fired at a rate > = 0.1 at any timestep during a trial. The text at the bottom of the plots indicates the number of PPC neurons that did not meet the criteria. The y-axis shows the magnitude of direction selectivity and the x-axis shows the angle of direction selectivity. The black vertical dashed lines represent the directions of the targets during the trials. a) FF. b) FB. c) LAT. d) FBLAT. The plots in a-d show that all model types evolved strong directionally selective PPC neurons for each of the trials (also see S5 and S7 Figs). (TIF) [file pone.0134669.s009.tif]

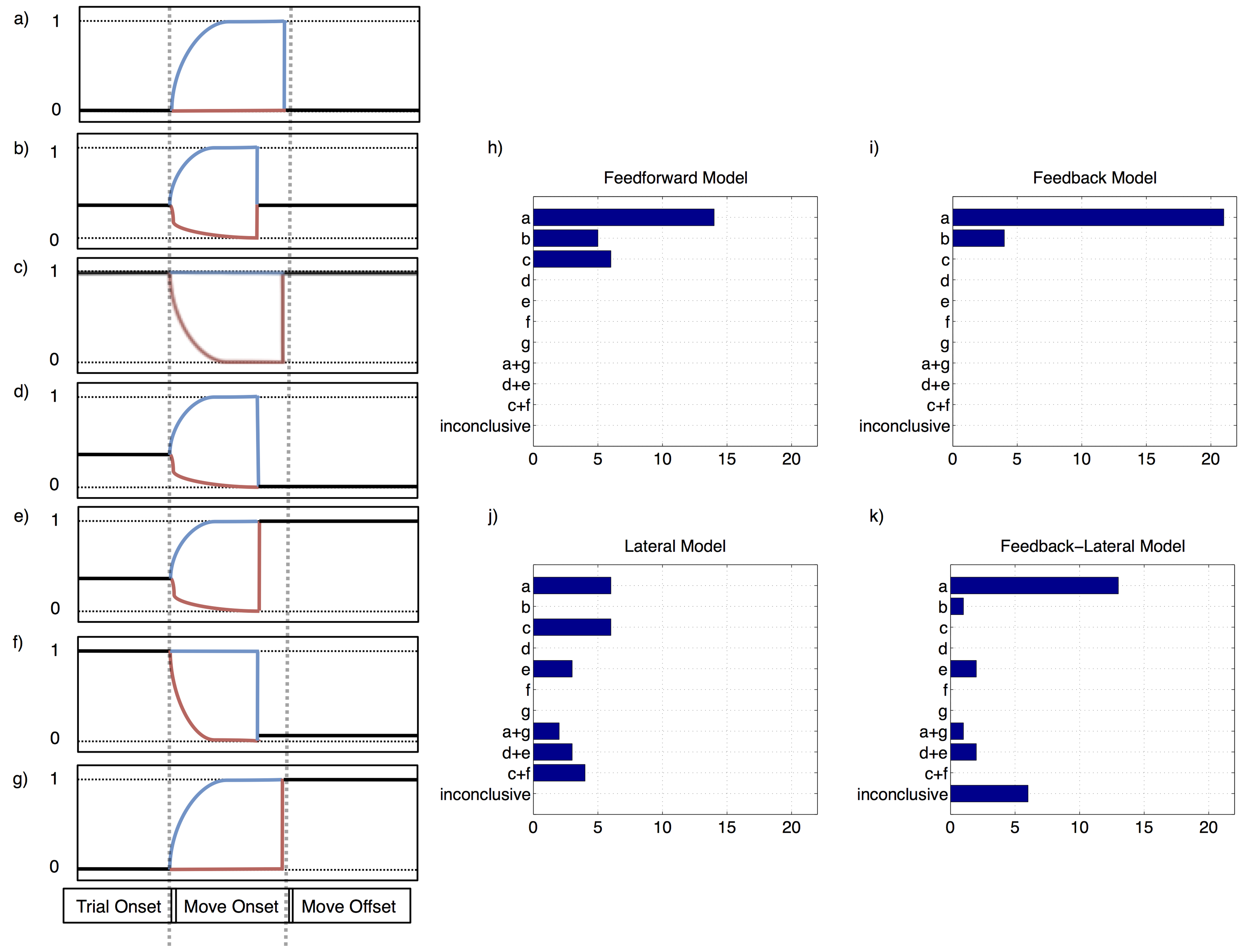

Supplement: S9 Fig — The schematic drawings in a-g represent the premotor/primary motor neural strategies for generating reaching trajectories to the correct targets across all trials for the 25 fittest agents of each model in the VG task. The y-axes of a-g represent the firing rate of opposing pairs of PMd/M1 neurons, which give rise to movement in all trials. The x-axes of a-g show the temporal progression of a trial (50 timesteps approximating 500ms). Neural strategy schematics a-g, are broken into three phases in temporal order; trial onset phase, movement onset phase, and movement offset phase. These three phases account for the PMd/M1 neural firing across the duration of the trial. The blue line represents the neural activity for the direction of hand movement (e.g., the Right neuron). The red line represents the neural activity for the direction counter to the hand’s trajectory towards the target (e.g., the Left neuron). The solid black lines represent an overlap of activity between the blue and red lines. With the exception of b, c, and e, all strategies either had initial activity near the minimum or maximum. In neural strategies b, d, and e the initial activity was in the range of [0.3, 0.9]. Plots h-k show histograms of the distributions of neural strategies used by the top 25 fittest agents per model. The combined strategies (a+g, d+e, and c+f) indicate that some agents had opposing pairs of neurons firing differently on all trials (e.g., the Up and Down neurons used a different strategy than the Right and Left neurons). The label ‘inconclusive’ in h-k, represent agents that did not perform well on all the trials yielding incorrect trajectories as a result of poorly defined neural strategies. a-g) PMd/M1 neural strategies for generating movements. h) Histogram of the Feedforward model’s top 25 agents’ neural strategies. i) Histogram of the Feedback model’s top 25 agents’ neural strategies. j) Histogram of the Lateral model’s top 25 agents’ neural strategies. k) Histogram of [file pone.0134669.s010.tif]
