# Supplementary material for: The Importance of Lateral Connections in the Parietal Cortex for Generating Motor Plans
Source: PLoS One. 2015 Aug 7;10(8):e0134669. doi: 10.1371/journal.pone.0134669 (PMC4529220; doi:10.1371/journal.pone.0134669)
Supplement: S1 Text — Evolutionary Algorithm section—details about the evolutionary algorithm utilized to tune free parameters in the simulation experiments; Fitness Comparisons section—comparisons of the models’ parameter evolution pertaining to the emergent behavior; Target Error Comparisons section—statistical comparisons of model accuracy in the two simulated reaching tasks (VG and MG); PPC Direction Selective Neurons section—description of the analysis and results showing how individual PPC neurons developed preferred direction selectivity, indicating their neural activation for only select trials; PMd/M1 Neural Strategies section—description of the procedure that distinguished between the different types of neural patterns in the output (PMd/M1) layer that led to good fitness values; Supplemental References section—list of references only cited in S1 Text sections. (DOCX) [file pone.0134669.s011.docx]

# Supplemental Information­­­

## Evolutionary Algorithm

Evolutionary algorithms are usually used to optimize a set of evolvable parameters (free parameters) by testing, selecting, altering and reproducing solutions until the parameters yield a desirable solution determined by a fitness function. An EA is a population-based metaheuristic optimization technique that utilizes mathematical operators inspired by biological evolution to implement mutation and reproduction (crossover or recombination). An EA is driven by an evaluation criterion (fitness function) used to find and select good solutions given some set of parameters. What makes EAs an attractive alternative to traditional a priori supervised learning algorithms (e.g. back-propagation), is that they ideally do not make any assumptions about the parameter space (akin to an unsupervised learning algorithm), which is advantageous when little is known about the parameters (e.g., the strength and type of connections within and between cortical regions). Removing the need for assumptions can allow for a more organic result, potentially leading to a better understanding of the underlying biology, and potential theoretical evidence for new phenomena.

In the field of artificial intelligence, evolutionary algorithms are often used to design and tune neural networks, which control agents or robots [[1-12](#_ENREF_1)]. In computational neuroscience, EAs have been used to fit electrophysiological recordings to the activity of neuron models [[13](#_ENREF_13),[14](#_ENREF_14)]. Most relevant to our work, neural networks were evolved to perform a reaching task with intentions of investigating the interaction between evolution and individual learning [[15](#_ENREF_15)]. In this simulation study however, the sensory inputs and neural architectures were abstract and not intended to investigate the mammalian brain or cortical interactions. More recently, neural networks were evolved in robotic platforms (neuro-robots) to investigate the relationship between selective attention and action selection by running experiments in a foraging task [[16](#_ENREF_16)]. Similar to our work, they investigated the relationship between different evolved architectures and the resulting behavior, but their neural architectures were not intended to model sensory integration or sensorimotor transformations, nor did their models provide any predictions pertaining to the underlying biology. Furthermore, these implementations did not constrain their neural network connectivity based on known neuroanatomy, and they did not attempt to evolve large parameter sets (up to 15613 parameters) that better reflect the intrinsic complexity of brain networks.

The EA used in the present experiments started with a population of 10 agents (generation 1) and grew to a population of 20 agents (generations 2-25000) through crossover. Mutations were applied to further alter the genes, allowing for faster searching of the parameter space. The best agent for every generation (1-25000) was passed to the next generation without being altered. After 25000 generations of evolution, the agent with the lowest fitness value (best fitness) was selected as the best agent. For each of the models, 100 independent evolutionary algorithms (EAs) were run, which resulted in 100 independently evolved best agents per model. Of the 100 best agents per model, the single agent that had the lowest (best) fitness value was deemed the champion agent after 25000 generations of evolution. The Evolving Objects library (http://eodev.sourceforge.net) was used to evolve the model’s free parameters. Keijzer and colleagues [[17](#_ENREF_17)] describe specifics of the library and algorithms implemented in their artificial evolution article.

The crossover and mutation operators allowed the EA to iteratively (through generations) manipulate the free parameters in a stochastic manner. The crossover operator took 2 parent agents’ free parameters, and mixed them together creating a single offspring agent. This process was repeated until 19 offspring agents were created per generation. All offspring agents had the same number of free parameters within the same bounding ranges as the parent agents per model. This implies that only the values of the free parameters are changed through crossover. The mutation operator had a static probability of mutation set to 0.4, which implied that an offspring agent had a 40% chance of undergoing mutations. Mutations were applied randomly (probability of 0.5) to all free parameters by the addition of a small random number drawn from a Gaussian distribution (G). In the case of the weights, these free parameters were bounded by [-1.0, 1.0] and the mutation values were drawn from G(0,0.3), with a mean of zero and a standard deviation of 0.3. For the sigmoid biases (each agent had independent PPC and PMd/M1 biases, i.e. 2 per agent), the values were bounded by [-5.0, 5.0] and the mutation values were drawn from G(0, 3). Lastly, for the sigmoid gains (2 per agent), the values were bounded by [0.1, 10.0] and the mutation values were drawn from G(0, 1.5).

## Fitness Comparisons

All four models found solutions to the reaching tasks, as measured by the agents’ fitness and accuracy (S2 and S4 Figs.). S2 Fig. shows the fitness evolution over generations. The red dashed line is the fitness of the champion agent and the black line shows the mean and standard deviation of the population. As will be discussed below, there were interesting and significant differences when comparing model performance in the VG and MG tasks.

The champion agents’ fitness, along with the mean and standard deviation of the population of best agents’ fitness, can be seen in the top 2 rows of Tables S1 for the VG task and rows 5-6 for the MG task. It can be seen that the champion agent per model (S4 Fig.: rows 1 and 5) achieved a fitness value at least 1 standard deviation lower than the mean (S4 Fig.: rows 2 and 6) of the respective populations in both tasks (compare S4 Fig.: row 1 to 2, and row 5 to 6). The population fitness values for the populations (all 100 of the best agents per model) show how well a model could evolve to perform either the VG (S3a Fig.) or the MG (S3b Fig.) task, and the level of behavioral diversity that evolved as a result of model complexity (Figure 1) and genome size. To see how well each of the models performed the respective tasks, compare the lowest data points between the models for each task (S3 Fig.). Whereas the Lateral model evolved the best fitness values in both tasks (S4 Fig.: rows 1 and 5), it also had a wider range of fitness values than FF and FB (S3a Fig.).

Lateral connections in the PPC were advantageous for visually guided movements, but had an even more prominent effect on memory guided movements. The LAT and FBLAT models outperformed the FF and FB models on MG movements (S3b Fig.). LAT also performed better than FF and FB on VG movements, but LAT had a wider range of fitness values (S3a Fig.).

The models’ median fitness values were compared using the Wilcoxon rank sum test for both the VG and MG task. For the VG task, the median fitness values for FF and FB models were significantly different (S5 Fig.: p-value = 1.47e^-10^), yet the pair wise comparisons between the LAT model and the FF or the FB models were not significantly different (S5 Fig.: p = 0.577 and p = 0.515 respectively). In addition, FF vs. FBLAT (p = 4.14e^-34^), FB vs. FBLAT (p = 1.38e^-28^), and LAT vs. FBLAT (p = 1.21e^-19^) were significantly different. These results suggest that performance by the LAT model, in terms of fitness, was comparable to the simpler FF model, and that the motor efference copy in the FB and FBLAT models was not necessarily advantageous. For the MG task, the LAT model was significantly better than all other models (S3b Fig.). All pair wise comparisons between LAT and the other three models using Wilcoxon rank sum tests were significant (S5 Fig.).

Taken together, these results suggest that accurate movements in the absence of sensory input (visual input from the target) require more complex connectivity within an area such as the parietal cortex and cannot be supported by a strictly feedforward architecture [[18](#_ENREF_18)]. Moreover, our results suggest that a feedback architecture carrying a motor efference copy is not sufficient for memory-guided movements.

## Target Error Comparisons

Rows 3-4 and 7-8 of S4 Fig. summarize performance of the different models in terms of target error (TE) on the respective tasks. TE was calculated as the average Euclidian distance in degrees of visual angle the hand was from the target location at the end of a trial, across all 8 trials per agent. In the VG task, all models demonstrated accurate movements (S4 Fig.: rows 3 and 4). In the MG task, lateral connections in the PPC were advantageous for accurate movements, in terms of target error (S4 Fig.: rows 7 and 8). As expected, TE increased when comparing VG to MG. But, performance was significantly worse in models without lateral connections.

The TE distributions for the champion agents and the population of agents show the models’ precision across all trials. To show the differences between the models’ TE, pair wise statistical comparisons were made with Wilcoxon rank sum tests for the medians of the distributions within each task. For the VG task, the champion agents TE medians reached significance when comparing FF to FB (p-value = 0.003, Bonferroni corrected for multiple comparisons) and FF to LAT (p-value = 1.55e^-4^), but none of the other comparisons reached significance (S5 Fig.: Visually Guided Task, Champion TE Medians). Similarly, for the champion agents in the MG task, only the TE median comparison between FF and LAT reached statistical significance (p-value = 0.005, Bonferroni corrected for multiple comparisons). For the median comparisons between the populations of agents TE in the VG task, only the median comparison between FB and LAT (p-value = 0.432) failed to reach significance, all other comparisons were highly significant (S5 Fig.: Visually Guided Task, Population TE Medians). In the MG task, the only median comparison that failed to reach significance was between FF and FB (p-value = 0.944); the other 5 comparisons were highly significant (S5 Fig.: Memory Guided Task, Population TE Medians).

Taken together, these results show that all models were accurate, based on target error, in the VG, but that lateral connections in the PPC were necessary for accurate performance in the MG task. Neither the Feedforward nor the Feedback models performed well in the MG task.

## PPC Direction Selective Neurons

PPC neurons showed strong direction selectivity for each model’s champion agent (S7 and S8 Figs.). The angles marked on the polar angle plots (S7 Fig.) depict the target locations or the angle of the reach vector needed to perform well in the respective trials.

PPC direction selectivity was determined for the champion agents of each model in the VG task. The firing rates for all 121 PPC neurons were analyzed individually to determine if they exhibited direction selectivity. Direction selectivity was determined for a PPC neuron by dividing its summed firing rate (when activity was above a 0.1 threshold across a trial) by the total number of timesteps its activity was above this threshold. This normalized firing rate was multiplied by the sine and cosine of the polar angle representing the different correct movement vectors (at 0, 45, 90, 135, 180, 225, 270, and 315 degrees). The summation of these vectors resulted in each PPC neuron’s direction selectivity index (S8 Fig.), where the magnitude of the vector sum was greatest if a PPC neuron fired predominately for one direction.

The PPC neurons shown in S8 Fig. indicate that the champion agents evolved direction selectivity for the target and non-target directions. The distributions of direction selective neurons for all models across the angles indicate that the agents evolved generalized representations of hand movements through space. In contrast, each model had several PPC neurons that were activated across all trials with very low firing rates, indicating a lack of direction selectivity (FF: 12; FB: 31; LAT: 35; FBLAT: 38). In addition, some neurons fired at a high rate for all trials (LAT: 11 and FBLAT: 2), also indicating a lack of direction selectivity (S8c and S8d Figs.: data points on the red dashed line). All models showed the strongest direction selectivity for the angles represented by the target locations (S8 Fig.: blue and black dashed lines respectively). The data show that the FF model’s champion agent had the greatest direction selectivity across its PPC neurons, likely due to the sensory driven nature of the architecture.

## PMd/M1 Neural Strategies

Several PMd/M1 neural strategies for generating movements evolved for the VG task, which resulted in significant behavioral diversity within and between the models. These neural strategies indicate different patterns of PMd/M1 neural firing rates across trials in the VG task. However, several of the evolved agents used PMd/M1 neural strategies that did not allow them to perform well on the respective tasks, so only the best 25 agents for each model were analyzed to interpret their neural strategies for movement generation. Empirical evidence supports the evolved PMd/M1 neural strategy variation in the models. PMd and M1 single unit recording studies in monkeys performing either a cursor moving task [[19](#_ENREF_19)] or a reaching task [[20](#_ENREF_20),[21](#_ENREF_21)], revealed that directionally tuned M1 and PMd neurons could either increase or decrease their firing rates upon initiation of hand movements in their preferred directions, This evidence indicates that our evolved neural strategies showed neural activity akin to both M1 and PMd neurons. Since our models contain 4 directionally encoded PMd/M1 neurons (see Fig. 1: Up, Down, Left, and Right), it was not possible to draw any conclusions about whether these neurons better represented premotor or primary motor cortex neurons. In fact, it appears that the diversity of evolved neural strategies in the VG task suggests that the simulated area could represent both neural populations.

Seven distinct PMd/M1 neural strategies were identified in the top 25 agents of each model (see S9a-S9g Figs.), and were defined by three phases of behavior (trial onset, movement onset, and movement offset). The trial onset or initial firing rate of the neurons is one major factor that defines the different strategies. The initial firing rate fell into one of three categories, low (i.e., firing rate < 0.3), medium (i.e., firing rate within [0.3, 0.9]), or high (i.e., firing rate > 0.9). The movement onset phase shows that movement was generated in 2 ways. Movements could be generated by an increased firing rate for a PMd/M1 neuron (or neurons in the case of diagonal movements) in the direction of intended movement (blue line of S9a, S9b, S9d, and S9g Figs.), or decreased firing rate of the PMd/M1 neuron (or neurons in the case of diagonal movements) in the direction opposite of intended movement (red line of S9c and S9f Figs.). The movement offset phase indicated that the pair (or both pairs in the case of diagonal movements) of opposing PMd/M1 neurons fired at the same rate again, as in the trial onset phase. However, the neural firing rates in the movement offset phase were dependent on the rates in the trial onset phase. This implies that an initial firing rate of 0.5 would result in a final firing rate of 0.5 for the neural strategy shown in S9b Fig. Although, all other neural strategies show that the PMd/M1 neural firing rates were either low or high, and usually 0 or 1 respectively.

Strategy a (S9a Fig.) was utilized by the majority of the top 25 agents for the FF, FB, and FBLAT models, and many of the LAT model’s agents (S9h-S9k Figs.). This particular strategy is stereotypical of neural activation, in that the neuron(s) encoded for the direction of movement, increased the firing rate(s) until the movement ceased, which at that point the neuron(s) also ceased firing. Similarly, neural strategies shown in S9b, S9d, S9e, and S9g Figs. depict increased neural activity for the direction of intended movement, the distinct differences between these strategies is demonstrated by the neural activity that terminated movement (S9b, S9d, S9e, and S9g Figs.: Move Offset phase). In contrast, neural strategies schematized in S9c and S9f Figs. depict movements made by lowered activity from the neuron(s) encoded for the direction opposite of intended movement (S9c and S9f Figs.: Move Onset). These neural strategies have also been shown for population averages of PMd neurons in reaching tasks [[20](#_ENREF_20),[21](#_ENREF_21)]. Therefore, it would appear that our directionally encoded PMd/M1 neurons sufficiently represented empirically reported neural activation in behaving monkeys.

Of the top 25 agents per model, the FF and FB models evolved only 3 and 2 different neural strategies respectively (S9h and S9i Figs.), whereas the LAT and FBLAT models showed more variety in their neural strategies (S9j and S9k Figs.). This variability of neural strategies gives rise to greater behavioral diversity, and may have resulted in better performance in the MG task. The inconclusive strategy (S9h-S9k Figs.: Inconclusive) implied that an agent did not evolve to perform the VG task well for all 8 targets. The FBLAT model evolved 6 of its best 25 agents with inconclusive strategies (S9k Fig.: Inconclusive), indicating that these 6 agents did not perform well in the VG task.

The evolved neural strategies provide insight as to how the agents generated movements to the targets in the VG task. Since the PPC firing rates drove the PMd/M1 neurons, it is important to investigate what kind of PPC neural firing patterns gave rise to these PMd/M1 neural strategies.

The neural firing patterns within the PPC ultimately drove the behavior via its projections to the PMd/M1 neurons. The PPC neurons displayed direction selectivity and topographic organization, which resulted in correct reaches to target locations in both tasks.

## Fitness Calculations

Training consisted of visually guided reaching to 8 targets from a center region over 25000 generations. During training, fitness values were calculated by summing the Euclidian distance in degrees of visual angle from an agent’s hand to the target, over all timesteps across all trials. This generated a single fitness value per agent that reflected both how quickly and accurately an agent moved its hand to the targets. The best (minimum) possible fitness value was 3563.4, which was the total amount of degrees of visual angle across all timesteps and trials that an agent would take to move to all 8 targets at the maximum velocity. Therefore, subtracting off 3563.4 from all agents’ fitness values provided a normalized fitness per agent that was optimized at 0 (S2 Fig.). The fitness calculation is given in Equation 5 (main text).

After training was complete (VG task), agents were tested in MG task that consisted of eight reaches to peripheral targets (S1 Fig.). The fitness function given in Equation 5 (main text) was used to evaluate each agent’s performance.

# Supplemental Figure Captions

**S1 Fig. Reaching Tasks.** The two tasks (Visually & Memory Guided Reach) are organized identically with eight potential peripheral target locations either 25° (vertical and horizontal targets) or 35° (diagonal targets) of visual angle away from the central position. Every trial for both tasks starts out with the fixation and hand aligned at the central position. For both tasks, one of the target locations is illuminated (provides visual input to the models) at the start of a trial, then for the visually guided task the target stays illuminated for the remainder of the trial, while the target disappears after 50ms (5 timesteps) for the memory guided task. The goal in each task is to keep fixation centered while moving the hand to the target location as quickly as possible and holding the hand at the target location for the remainder of the trial.

**S2 Fig. Evolution of Fitness.** Each plot shows the evolution of the fitness values for the best agents of 100 independent evolutionary algorithms (EAs) (supplement to Fig. 2). Fitness values were minimized and corrected, which indicates that the best possible fitness value was 0. The black line is the mean fitness of all 100 agents with the width of the line showing the standard deviation around the mean at every generation. The red dashed line is the evolution of fitness for the champion agent at the last generation. The y-axes show the corrected fitness values calculated from the summed Euclidian distance in degrees of visual angle between the hand and the target for every timestep across all trials. The x-axes show the generation number, going from 1 to 25000. a) Evolution of fitness values for the FF model. b) Evolution of fitness values for the FB model. c) Evolution of fitness values for the LAT model. d) Evolution of fitness values for the FBLAT model.

**S3 Fig. Population Fitness.** The fitness values of the 100 agents after 25000 generations of evolution is supplemental to Fig. 3. Each data point (black dot) represents a single agent’s fitness value. The y-axes show the fitness values calculated for the tasks, with the scales set (VG: 0-2500; MG; 0-6000) to illuminate the differences between the models. The x-axes show the different models (FF: Feedforward, FB: Feedback, LAT: Lateral, FBLAT: Feedback-Lateral). a) Population fitness values for the 100 best agents in the visually guided (VG) task. b) Population fitness values for the 100 best agents in the memory guided (MG) task.

**S4 Fig. Fitness and Target Error: VG and MG Tasks.** The fitness values are the summed Euclidian distance from the target at every timestep across all 8 trials. The fitness values were corrected by subtracting off the minimum possible fitness value for each trial to make the best possible fitness value equal to 0. The target error, which is given in degrees of visual angle, is the average Euclidian distance and standard deviation from the target across the trials, with the minimum target error equal to 0. The columns show the data from the four models (from left to right: FF: Feedforward, FB: Feedback, LAT: Lateral, FBLAT: Feedback-Lateral). The champion agent had the best fitness of the 100 independently evolved agents per model. The top four rows (rows 1 - 4) represent data from the VG task and the last four rows (rows 5 - 8) represent data from the MG task (supplement to Fig. 2).

**S5 Fig. p-Value Comparisons.** All data shown, reflect p-values calculated with Wilcoxon rank sum tests of pair wise comparisons between medians of data from the models labeled in the 1^st^ column (FF: Feedforward model; FB: Feedback model; LAT: Lateral model; FBLAT: Feedback-Lateral model). Bold values indicate significance (α = 0.05, p < 0.008 Bonferroni corrected for multiple comparisons). The labeled columns (Fitness Medians; Population TE Medians; Champion TE Medians) for the 2 tasks (Visually Guided and Memory Guided) indicate the pair wise comparison between the medians from the distribution of fitness values for all 100 agents per model, the medians from the distribution of target error (TE) for the population of 100 agents per model, and the medians from the distribution of TE for only the champion agents per model (supplement to Fig. 3).

**S6 Fig. Reaching Trajectories and Velocity Profiles with Different Sensory Weights (FF and LAT Models).** The data shown are comparable to that shown in Figs. 2 and 3 for the Feedforward (FF) and Lateral (LAT) models. The difference, is the sensory input weight scale factor (*f*) is set to +4 for Vision and+4 for Proprioception (see Equation 1; *f* parameter) to contrast the subtractive weight data shown in Figs. 2 and 3 (Equation 1; *f* set to +2 for Vision and -4 for Proprioception). The first two columns show reaching trajectories for the FF (column 1) and LAT (column 2) models similar to Fig. 2. The third and fourth columns show the average velocity profile for the FF and LAT models respectively, similar to Fig. 3. a) The champion agents’ reaching trajectories (FF: column 1; LAT: column 2) and velocity profiles (FF: column 3; LAT: column 4) for the VG task. b) The means and standard error of the means (SEMs) for reaching trajectories (FF: column 1; LAT: column 2) and velocity profiles (FF: column 3; LAT: column 4) for the VG task. c) The champion agents’ reaching trajectories (FF: column 1; LAT: column 2) and velocity profiles (FF: column 3; LAT: column 4) for the MG task. d) The population means and SEMs for reaching trajectories (FF: column 1; LAT: column 2) and velocity profiles (FF: column 3; LAT: column 4) for the MG task.

**S7 Fig. PPC Neural Direction Selectivity.** Representative directionally selective PPC neurons that supplement the data shown in Fig. 4. The polar angle plots depict the normalized firing rate of different PPC neurons corresponding to different directions of movement. The central plots depict exemplars of non-directionally selective neurons. a) FF. b) FB. c) LAT. d) FBLAT.

**S8 Fig. Direction Selectivity Index.** The top plots of a-d show the direction selectivity index for all PPC neurons that fired at a rate >= 0.1 at any timestep during a trial. The text at the bottom of the plots indicates the number of PPC neurons that did not meet the criteria. The y-axis shows the magnitude of direction selectivity and the x-axis shows the angle of direction selectivity. The black vertical dashed lines represent the directions of the targets during the trials. a) FF. b) FB. c) LAT. d) FBLAT. The plots in a-d show that all model types evolved strong directionally selective PPC neurons for each of the trials (also see S5 and S7 Figs.).

**S9 Fig. PMd/M1 Neural Strategies for the Top 25 Fittest Agents.** The schematic drawings in a-g represent the premotor/primary motor neural strategies for generating reaching trajectories to the correct targets across all trials for the 25 fittest agents of each model in the VG task. The y-axes of a-g represent the firing rate of opposing pairs of PMd/M1 neurons, which give rise to movement in all trials. The x-axes of a-g show the temporal progression of a trial (50 timesteps approximating 500ms). Neural strategy schematics a-g, are broken into three phases in temporal order; trial onset phase, movement onset phase, and movement offset phase. These three phases account for the PMd/M1 neural firing across the duration of the trial. The blue line represents the neural activity for the direction of hand movement (e.g., the Right neuron). The red line represents the neural activity for the direction counter to the hand’s trajectory towards the target (e.g., the Left neuron). The solid black lines represent an overlap of activity between the blue and red lines. With the exception of b, c, and e, all strategies either had initial activity near the minimum or maximum. In neural strategies b, d, and e the initial activity was in the range of [0.3, 0.9]. Plots h-k show histograms of the distributions of neural strategies used by the top 25 fittest agents per model. The combined strategies (a+g, d+e, and c+f) indicate that some agents had opposing pairs of neurons firing differently on all trials (e.g., the Up and Down neurons used a different strategy than the Right and Left neurons). The label ‘inconclusive’ in h-k, represent agents that did not perform well on all the trials yielding incorrect trajectories as a result of poorly defined neural strategies. a-g) PMd/M1 neural strategies for generating movements. h) Histogram of the Feedforward model’s top 25 agents’ neural strategies. i) Histogram of the Feedback model’s top 25 agents’ neural strategies. j) Histogram of the Lateral model’s top 25 agents’ neural strategies. k) Histogram of the Feedforward model’s top 25 agents’ neural strategies. The plots in h-k show that the more complex models evolved a more diverse strategy distribution (compare j and k to h and i).

# Supplemental References

1. Bongard J. Behaviour Chaining: Incremental Behaviour Integration for Evolutionary Robotics; 2008; Winchester, UK.

2. Bongard J, Pfeifer R (2003) Evolving Complete Agents using Artificial Ontogeny. Morpho-functional Machines: The New Species (Designing Embodied Intelligence): 237-258.

3. Dellaert NP, Melo MT (1996) Stochastic lot-sizing: Solution and heuristic methods. Int J Prod Econ 46: 261-276.

4. Floreano D, D\"u r, Peter, Mattiussi C (2008) Neuroevolution: from architectures to learning. Evolutionary Intelligence 1: 47-62.

5. Floreano D, Husbands P, Nolfi S (2008) Evolutionary Robotics. In: Siciliano B, Khatib O, editors. Springer Handbook of Robotics, Chapter 61: Springer.

6. Floreano D, Mattiussi C (2001) Evolution of Spiking Neural Controllers for Autonomous Vision-Based Robots. Proceedings of the international Symposium on Evolutionary Robotics From intelligent Robotics To Artificial Life 2217, T. Gomi, Ed. Lecture Notes In Computer Science Springer-Verlag, London, 38-61.

7. Izquierdo SS, Izquierdo LR (2013) Stochastic Approximation to Understand Simple Simulation Models. J Stat Phys 151: 254-276.

8. Mouret J-B, Doncieux S. Incremental Evolution of Animats' Behaviors as a Multi-objective Optimization. In: Asada M, et al., editors; 2008; Osaka, Japan. Springer. pp. 210-219.

9. Oros N, Steuber V, Davey N, Cañamero L, Adams R. Evolution of bilateral symmetry in agents controlled by spiking neural networks; 2009 3 2009-April 2. pp. 116-123.

10. Stanley KO, D'Ambrosio DB, Gauci J (2009) A hypercube-based encoding for evolving large-scale neural networks. Artif Life 15: 185-212.

11. Stanley KO, Miikkulainen R (2003) A Taxonomy for Artificial Embryogeny. Artificial Life journal 9: 93-130.

12. Stanley KO, Miikkulainen R (2002) Evolving neural networks through augmenting topologies. Evol Comput 10: 99-127.

13. Rossant C, Leijon S, Magnusson AK, Brette R (2011) Sensitivity of Noisy Neurons to Coincident Inputs. Journal of Neuroscience 31: 17193-17206.

14. Van Geit W, Achard P, De Schutter E (2007) Neurofitter: a parameter tuning package for a wide range of electrophysiological neuron models. Front Neuroinform 1: 1.

15. Cecconi F, Parisi D (1990) Evolving organisms that can reach for objects. Proceedings of the first international conference on simulation of adaptive behavior on From animals to animats. Paris, France: MIT Press. pp. 391-399.

16. Petrosino G, Parisi D, Nolfi S (2013) Selective attention enables action selection: evidence from evolutionary robotics experiments. Adaptive Behavior.

17. Keijzer M, Merelo JJ, Romero G, Schoenauer M (2002) Evolving objects: A general purpose evolutionary computation library. Lect Notes Comput Sc 2310: 231-242.

18. Matsumoto R, Nair DR, Ikeda A, Fumuro T, LaPresto E, et al. (2012) Parieto-frontal network in humans studied by cortico-cortical evoked potential. Human Brain Mapping 33: 2856-2872.

19. Cisek P (2002) Neural Activity in Primary Motor and Dorsal Premotor Cortex In Reaching Tasks With the Contralateral Versus Ipsilateral Arm. Journal of Neurophysiology 89: 922-942.

20. Crammond DJ, Kalaska JF (1996) Differential relation of discharge in primary motor cortex and premotor cortex to movements versus actively maintained postures during a reaching task. Exp Brain Res 108: 45-61.

21. Rickert J, Riehle A, Aertsen A, Rotter S, Nawrot MP (2009) Dynamic Encoding of Movement Direction in Motor Cortical Neurons. Journal of Neuroscience 29: 13870-13882.
